# Immunity and other defenses in pea aphids, *Acyrthosiphon pisum*

**DOI:** 10.1186/gb-2010-11-2-r21

**Published:** 2010-02-23

**Authors:** Nicole M Gerardo, Boran Altincicek, Caroline Anselme, Hagop Atamian, Seth M Barribeau, Martin de Vos, Elizabeth J Duncan, Jay D Evans, Toni Gabaldón, Murad Ghanim, Adelaziz Heddi, Isgouhi Kaloshian, Amparo Latorre, Andres Moya, Atsushi Nakabachi, Benjamin J Parker, Vincente Pérez-Brocal, Miguel Pignatelli, Yvan Rahbé, John S Ramsey, Chelsea J Spragg, Javier Tamames, Daniel Tamarit, Cecilia Tamborindeguy, Caroline Vincent-Monegat, Andreas Vilcinskas

**Affiliations:** 1 Department of Biology, Emory University, O Wayne Rollins Research Center, 1510 E. Clifton Road NE, Atlanta, GA, 30322, USA; 2 Interdisciplinary Research Center, Institute of Phytopathology and Applied Zoology, Justus-Liebig-University of Giessen, Heinrich-Buff-Ring 26-32, D-35392 Giessen, Germany; 3 Université de Lyon, INRA, INSA-Lyon, IFR41 BioEnvironnement et Santé, UMR203 BF2I, Biologie Fonctionnelle Insectes et Interactions, Bat. Louis-Pasteur 20 ave Albert-Einstein, F-69621 Villeurbanne, France; 4 UMR Interactions Biotiques et Santé Végétale, INRA 1301-CNRS 6243-Université de Nice-Sophia Antipolis, 400 routes des Chappe, F-06903 Sophia-Antipolis cedex, France; 5 Department of Nematology, Graduate Program in Genetics, Genomics and Bioinformatics, University of California, 900 University Ave, Riverside, CA 92521, USA; 6 Boyce Thompson Institute for Plant Research, Ithaca, NY 14853, USA; 7 Genetics Otago and The Laboratory for Evolution and Development, Department of Biochemistry, University of Otago, Box 56, Dunedin 9054, New Zealand; 8 USDA-ARS Bee Research Lab, BARC-East Bldg 476, Beltsville, MD 20705, USA; 9 Bioinformatics and Genomics Programme, Centre for Genomic Regulation (CRG), Doctor Aiguader 88, 08003 Barcelona, Spain; 10 Department of Entomology, The Volcani Center, Bet Dagan 50250, Israel; 11 Instituto Cavanilles de Biodiversidad y Biología Evolutiva, Universitat de València, Avenida Blasco Ibañez 13, 46071 València, Spain; 12 CIBER en Epidemiología y Salud Pública (CIBEResp) and Centro Superior de Investigación en Salud Pública (CSISP), Conselleria de Sanidad (Generalitat Valenciana), Avenida de Cataluña 21, 46020 València, Spain; 13 Advanced Science Institute, RIKEN, 2-1 Hirosawa, Wako, Saitama 351-0198, Japan; 14 Plant Pathology and Plant-Microbe Biology Department, Cornell University, Tower Road, Ithaca, NY 14853, USA; 15 Department of Entomology, Texas A&M, College Station, TX 77843-2475, USA

## Abstract

**Background:**

Recent genomic analyses of arthropod defense mechanisms suggest conservation of key elements underlying responses to pathogens, parasites and stresses. At the center of pathogen-induced immune responses are signaling pathways triggered by the recognition of fungal, bacterial and viral signatures. These pathways result in the production of response molecules, such as antimicrobial peptides and lysozymes, which degrade or destroy invaders. Using the recently sequenced genome of the pea aphid (*Acyrthosiphon pisum*), we conducted the first extensive annotation of the immune and stress gene repertoire of a hemipterous insect, which is phylogenetically distantly related to previously characterized insects models.

**Results:**

Strikingly, pea aphids appear to be missing genes present in insect genomes characterized to date and thought critical for recognition, signaling and killing of microbes. In line with results of gene annotation, experimental analyses designed to characterize immune response through the isolation of RNA transcripts and proteins from immune-challenged pea aphids uncovered few immune-related products. Gene expression studies, however, indicated some expression of immune and stress-related genes.

**Conclusions:**

The absence of genes suspected to be essential for the insect immune response suggests that the traditional view of insect immunity may not be as broadly applicable as once thought. The limitations of the aphid immune system may be representative of a broad range of insects, or may be aphid specific. We suggest that several aspects of the aphid life style, such as their association with microbial symbionts, could facilitate survival without strong immune protection.

## Abstract 2

The genome of the pea aphid *Acyrthosiphon pisum* lacks genes thought to be crucial in other insects for recognition, signaling and killing of microbes.

## Background

Aphids face numerous environmental challenges, including infection by diverse pathogens and parasites. These pressures include parasitoid wasps, which consume their hosts as they develop inside, and a variety of viral, bacterial and fungal pathogens. Both parasitoid wasp and fungal pathogens cause significant decline of natural aphid populations [1,2], and have been suggested as potential agents for biocontrol of these agriculturally destructive pests. While facing such challenges, aphids also cope with predators and abiotic stresses, such as extreme temperature fluctuations. Thus, like most insects, aphids must attempt to survive in a harsh, complex environment.

Insects have a number of defense mechanisms. First, many insects, including aphids, behaviorally avoid predators, pathogens, and environmental stressors [3-6]. When stressors cannot be avoided, insects have a protective cuticle and gut pH inhospitable to many foreign organisms. If these barriers fail, immunological defense mechanisms recognize the invader, triggering a signaling cascade and response. While insects do not have adaptive, antigen-based responses typical of vertebrates, insects do have innate immune responses, which include clotting, phagocytosis, encapsulation, and production of antimicrobial substances [7,8]. Phagocytosis and encapsulation are referred to as cellular responses as they are mediated by blood cells [9]. Reponses vary depending on the invader, with antimicrobial peptides being central to combating microbes and encapsulation being central to combating larger invaders, such as parasitoids. Until recently, it was presumed that insects were limited to these non-specific innate immune responses and had no specific immunity (for example, the antigen-based immune response of humans). There is, however, increasing evidence for the ability of insects to mount specific immune responses [10].

Here we focus on the identification of aphid genes that are known to play a role in the recognition and degradation of microbial pathogens in other insects, as these are the invertebrate defense processes that are best understood. In the fruit fly *Drosophila melanogaster*, recognition of an invasive microbe leads to signal production via four pathways (Toll, immunodeficiency (IMD), c-Jun N-terminal kinase (JNK), and Janus kinase/Signal transducers and activators of transcription (JAK/STAT)) [11]. Each pathway is activated in response to particular pathogens [12]. Signaling triggers the production of a multitude of effectors, including, most notably, antimicrobial peptides (AMPs). Insect AMPs may be 1,000-fold induced in microbe-challenged insects compared to basal levels. In insect genomes annotated to date, these pathways appear well conserved, with most of the key components found across flies (*Drosophila *spp.), mosquitoes (*Aedes aegypti*, *Anopheles gambiae*), bees (*Apis mellifera*) and beetles (*Tribolium castaneum*) [13-17].

Because aphids and other insects face diverse challenges, we propose models for several genes critical to other elements of insect stress responses. These include genes encoding heat shock proteins (HSPs), which are synthesized in almost all living organisms when exposed to high temperatures or stress [18]. We also suggest models for genes involved in the synthesis of the alarm pheromone (*E*)-β farnesene, which aphids release in the presence of predators [19]. While there are undoubtedly many other genes involved in stress and immunological responses, our selection of genes for exploration provides a broad survey of the known insect immune and stress repertoire and will serve as a basis for future exploration of more specific responses.

The pea aphid genome provides novel insights into arthropod immunity for two reasons. First, most of our understanding of insect immune and stress responses comes from holometabolous insects, the group of insects with complete metamorphisis, such as flies, butterflies, beetles and bees. The genome of the hemimetabolous pea aphid, *Acyrthosiphon pisum*, may thus provide novel insight into immunity and defense in more basal, non-holometabolous insects, which have incomplete metamorphisis. Second, aphids are unique amongst the arthropods sequenced to date in that they are intimately dependent on both obligate and facultative bacterial symbionts for their survival. The aphid symbiont community includes *Buchnera aphidicola*, obligate and intracellular Gram-negative bacteria that have the ability to synthesize required amino acids not readily available in the aphid diet. Beyond this obligate symbiosis, aphids frequently host one or more additional Gram-negative bacterial symbionts, including most notably *Hamiltonella defensa*, *Serratia symbiotica *and *Regiella insecticola *[20,21]. Unlike *Buchnera*, which is present in all aphids and is thus considered a primary symbiont, these bacteria are considered to be facultative, secondary symbionts, because their presence varies within an aphid species [22]. Secondary symbiotic bacteria have been shown to influence several aspects of aphid ecology, including heat tolerance and resistance to parasites and pathogens [23-26]. Specifically, both *H. defensa *and *S. symbiotica *confer protection against parasitoid wasp development [27,28], and *R. insecticola *decreases *A. pisum *mortality after exposure to the fungal pathogen *Pandora neoaphidis *[29]. These are some of the best-studied examples of symbiont-conferred protection [30].

Aphids thus provide an excellent opportunity to study the immune system of an organism that is dependent on microbial symbionts but is hampered by parasites and pathogens. Despite this, little work has been done to characterize the aphid immune response. Altincicek *et al. *[31] found that compared to other insects, stabbing a pea aphid with bacteria elicits reduced lysozyme-like (muramidase) activity, and no detectable activity against live bacteria in hemolymph assays. Furthermore, suppression subtraction hybridization (SSH) of bacterial-challenged aphids uncovered no antimicrobial peptides and few genes of known immune function [31]. These results are surprising given that similar studies in other insects demonstrate that antimicrobial peptide production and upregulation of immune-related genes is a common feature of the insect immune response that can be captured in functional assays such as SSH [32-35]. This suggests that aphids have a significantly reduced or altered immune repertoire.

Using the recently sequenced genome of the pea aphid clone LSR1, in this study, we take two approaches to study immunity and stress in pea aphids. First, we assay presence/absence of a subset of known immune and stress-related genes. Second, we combine functional assays targeting the production of RNA and proteins to gain insight into how pea aphids respond to various challenges. Overall, our results suggest that pea aphids are missing many genes central to immune function in other insects, and that, although pea aphids do mount some response to challenges, the overall immune-response of pea aphids is more limited than that of other insects studied to date.

## Results and discussion

### Overview of annotation

We focused our manual annotation efforts on a subset of genes involved in the innate, humoral immune response contributing to recognition, signaling and response to bacteria and fungi in arthropods. We also manually annotated some genes involved in more general stress responses (for example, HSPs). All annotations are based on the recently completed sequencing of pea aphid clone LSR1 [36]. All genes manually annotated, as well as those genes that we found to be missing in the pea aphid genome, are listed in Table S1 in Additional file 1. Also in this table, BLAST-based searches revealed that another aphid, *Myzus persicae *(green peach aphid), has putative homologs for many immune and stress related genes identified in the pea aphid.

### Annotation of microbial recognition genes

#### Peptidoglycan receptor proteins

Upon microbial invasion, *Drosophila *utilize several pathogen recognition receptors (PRRs) to detect pathogen-specific molecular patterns (for example, cell-surface motifs) [37]. PRRs include peptidoglycan receptor proteins (PGRPs), which recognize peptidoglycans present in cell walls of Gram-positive and Gram-negative bacteria. PGRP-based recognition activates both the Toll and IMD/JNK pathways. PGRPs are highly conserved, with mammals and insect PGRPs sharing a 160 amino acid domain [38,39]. Thus, it is surprising that pea aphids, in contrast to all other sequenced insects, appear to have no PGRPs. One other sequenced arthropod, the crustacean *Daphia pulex*, is also missing PGRPs [40].

#### Gram-negative binding proteins

GNBPs (Gram-negative binding proteins, a historical misnomer) are thought to detect Gram-positive bacteria [41]. GNBPs and PGRPs are suspected to form a complex. GNBPs then hydrolyze Gram-positive peptidoglycans into small fragments, which are detected by PGRPs [41,42]. Aphids have two *GNBP *paralogs, *GNBP1 *and *GNBP2 *(see Figure S1a in Additional file 1). Because GNBPs are thought to form a complex with PGRPs, the presence of GNBPs without PGRPs in aphids, as well as in the crustacean *D. pulex *[40], calls into question whether GNBPs play a role in bacterial detection in these organisms. Some GNBPs and similar proteins are known to function in fungal recognition [42], which may be the primary function of these molecules in aphids.

#### Lectins

Lectins are a diverse group of sugar binding proteins. Many lectins function in insect immune recognition by binding to polysaccharide chains on the surface of pathogens [43]. *Drosophila *c-type lectins also appear to facilitate encapsulation of parasitoid invaders, by marking surfaces for hemocyte recruitment [44]. Aphids have five *c-type lectin *paralogs.

Galectins are another widely-distributed group of lectins [45]. In mosquitoes, *galectins *are upregulated in response to both bacterial and malaria parasite infection [46,47]. Insect galectins are thought to be involved in either pathogen recognition, via recognition of β-galactoside, or in phagocytosis [45]. Aphids have two *galectin *paralogs.

#### Class C scavenger receptors

In *Drosophila*, Scavenger receptors exhibit broad affinity towards both Gram-positive and Gram-negative bacteria, but not yeast [48]. Pathogen recognition by class C scavenger receptors in *Drosophila *facilitates phagocytosis, and natural genetic variation of *Drosophila *scavenger receptors is correlated with variation in the ability to suppress bacterial infection [49]. While *D. melanogaster *has four class C scavenger receptor homologs, *A. gambiae *and *A. mellifera *have only one. Pea aphids appear to have no class C scavenger receptors.

#### The Nimrod superfamily and Dscam

Several members of the Nimrod superfamily appear to function as receptors in phagocytosis and bacterial binding [50,51]. Such insect genes include *eater *and *nimrod*. Many of these genes are characterized by a specific EGF (epidermal growth factor) repeat, and are duplicated in the genomes of *D. melanogaster*, *T. castaneum *and *A. mellifera *[52]. We were unable to identify any EGF motif genes in the pea aphid genome.

Complex alternative splicing of Dscam (Down syndrome cell adhesion molecule) generates diverse surface receptors sometimes employed in arthropod innate immune defenses [53-55]. Though we did not manually annotate this complex gene as a part of this initial aphid immune gene project, we did identify multiple predicted protein sequences coded by the aphid genome with strong similarity to Dscam in other insects [GenBank: XP_001951010, XP_001949262, XP_001945921, XP_001951684, XP_001942542]. Further investigations will be necessary to determine the activity and hypervariability of these genes and their transcripts in aphids.

### Annotation of signaling pathways

#### The Toll signaling pathway

**Figure 1:**
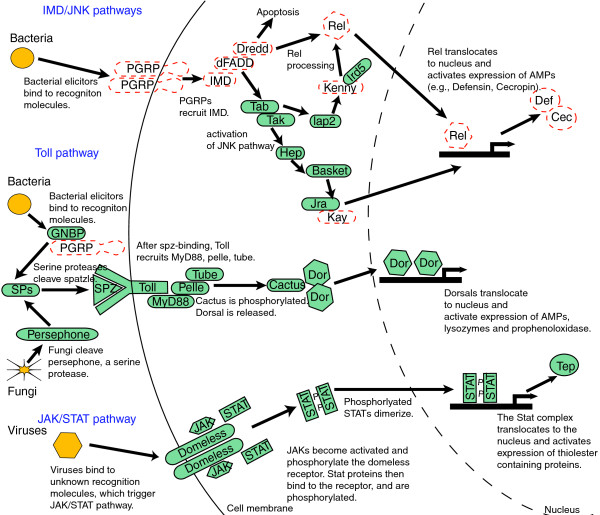
**Some key insect recognition, signaling and response genes are missing in the pea aphid**. Previously sequenced genomes of other insects (flies, mosquitoes, bees, beetles) have indicated that immune signaling pathways, seen here, are conserved across insects. In aphids, missing IMD pathway members (dashed lines) include those involved in recognition (PGRPs) and signaling (IMD, dFADD, Dredd, REL). Genes encoding antimicrobial peptides common in other insects, including defensins and cecropins, are also missing. In contrast, we found putative homologs for most genes central to the Toll, JNK and JAK/STAT signaling pathways.

The Toll pathway is a signaling cascade involved in both development and innate immunity. In *Drosophila*, deletion of many of the component genes leads to increased susceptibility to many Gram-positive bacteria and fungal pathogens [11], and some Gram-negative bacteria and viruses [12]. In addition, upregulation of many components of the Toll pathway is observed following parasitoid wasp invasion [56]. The Toll pathway appears to be intact in pea aphids. We found convincing matches for genes encoding the extracellular cytokine spätzle, the transmembrane receptor Toll, the tube and MyD88 adaptors, the kinase pelle, the inhibitor molecule cactus (a homolog of IkB), cactin, Pellino, Traf, and the transactivator dorsal (Figure 1). The latter two genes are duplicated.

As in other insects, there are several gene families associated with the Toll pathway that are represented in aphids. First, aphids seem to have multiple *spätzles *that segregate with *Drosophila spätzles *1, 2, 3, 4 and 6 in phylogenetic analyses (Figure S1b in Additional file 1). Second, aphids also have a suite of serine proteases and serine protease inhibitors (serpins). Though we did not manually annotate serine proteases and serpins as a part of this initial aphid immune gene project, we did identify multiple predicted protein sequences in the aphid genome with strong similarity to serine proteases and serpins in other insects. In insects, these molecules function in digestion, embryonic development and defense responses towards both microbial and parasitoid wasp invaders [57-59]. In the absence of microbial challenge, the serpin necrotic prevents activation of the Toll pathway, but upon immunological challenge, the Toll pathway is triggered by a cascade of serine proteases, including persephone, which is thought to be specific to fungal challenge [41]. Though it is not clear which of the many aphid serine proteases is homologous to persephone, it is likely that pea aphids have serine proteases capable of triggering the Toll pathway. Finally, aphids also have multiple genes encoding Toll receptors, which function as transmembrane receptors in both mammals and insects. While nine single-copy Toll genes have been identified in *D. melanogaster *(*Toll1 *to *Toll9*), it seems that pea aphids, like other insects, lack some of these genes, but have multiple copies of others (Figure S1c in Additional file 1). In other organisms, some, but not all, Tolls serve a role in immune function, while others function in developmental processes [60-62]. For aphids, it is not yet clear what role each Toll serves.

#### The JAK/STAT signaling pathway

Like the Toll pathway, in *Drosophila*, the JAK/STAT pathway is involved in both development and immunity. The JAK/STAT pathway is the least understood of the core insect immune pathways. JAK/STAT pathway induction appears to lead to overproliferation of hemocytes, upregulation of thiolester-containing proteins (TEPs), and an antiviral response [63]. Changes in gene expression following parasitoid wasp invasion of *Drosophila *larvae suggest a role for the JAK/STAT pathway in parasitoid response [56]. Pea aphids have homologs of all core JAK/STAT genes, including genes encoding the cytokine receptor domeless, JAK tyrosine kinase (aka Hopscotch), and the STAT92E transcription factor (Figure 1). *STAT92E *appears to be duplicated. No homologs were found for *upd *(unpaired), considered a key ligand in *Drosophila *JAK/STAT induction. This ligand is also missing in other insects (for example, *A. mellifera*) [14].

#### IMD and JNK signaling pathways

**Figure 2:**
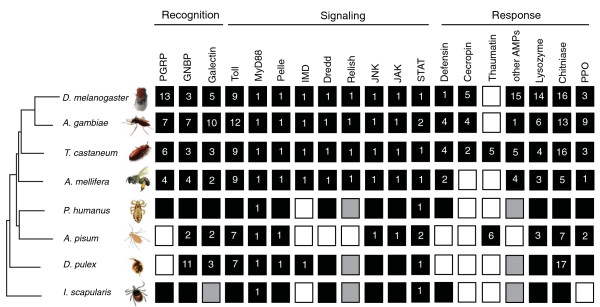
**Gene families implicated in arthropod immunity suggest unique features of the pea aphid immune system**. Black indicates present (copy number is indicated, when known), white indicates absent, and gray indicates equivocal or unknown. Values for *D. melanogaster*, *A. gambiae*, *T. castanateum*, *A. mellifera*, and some *D. pulex *genes are based on published analyses [13,14,16,17,40]. For previously unannotated *D. pulex *genes, as well as for *I. scapularis *and *P. humanus *genes, we determined presence via cursory BLAST searches against available genome databases [127,128] (wfleabase.org, vectorbase.org) using both *D. melanogaster *and *A. pisum *protein sequences as queries. Gene presence for *Ixodes *was confirmed based on previous studies [129]. Future comprehensive annotation of the *Pedicularis *and *Ixodes *immune gene sets may reveal the presence of additional genes and lack of functionality of others. PPO, prophenoloxidase.

Surprisingly, pea aphids appear to be missing many crucial components of the IMD signaling pathway. This pathway is critical for fighting Gram-negative bacteria in *Drosophila *[11,64], and IMD pathway member knockouts influence susceptibility to some Gram-positive bacteria and fungi as well [12]. IMD-associated genes missing in pea aphids include *PGRP*s (see above), *IMD*, *dFADD*, *Dredd *and *Relish *(*Rel*) (Figure 1). In contrast, conserved one-to-one orthologs of these same genes are found across *Drosophila*, *Apis*, *Aedes*, *Anopheles *and *Tribolium *[13]. Cursory BLAST-based searches for these genes in other arthropods suggest that some may be missing (Figure 2). Pea aphids do have homologs for a few pathway members (*TAB*, *TAK*, *kenny*, *Iap2 *and *IRD5*; Figure 1).

While missing IMD-associated genes, pea aphids have plausible orthologs for most components of the JNK pathway (Figure 1). In *Drosophila*, the JNK pathway regulates many developmental processes, as well as wound healing [65], and has been proposed to play a role in antimicrobial peptide gene expression and cellular immune responses [11,66]. Genes present include *hep*, *basket*, and *JRA*. Searches for homologs to the *Drosophila kayak* (*kay*) gene found an apparently similar transcription factor encoding gene in the *A. pisum* genome [GenBank: XP_001949014], but this match was largely restricted to the leucine zipper region, and failed tests of reciprocity.

The absence of IMD but presence of JNK in pea aphids is surprising as, in *Drosophila*, the IMD signaling pathway leads to activation of components of the JNK signaling pathway [11]. Specifically, when TAK, a protein kinase of the IMD pathway, is activated, it triggers the JNK pathway. Whether TAK can be activated without the rest of the IMD pathway is unknown. An alternative IMD-independent activation of JNK, via the inducer Eiger [67], has been proposed in *Drosophila *[66]. As *Eiger *is present in the pea aphid, this mode of activation may serve a critical role in any aphid JNK-based immune response.

### Annotation of recognition genes

#### Antimicrobial peptides

Introduction of microbes into most insects leads to the production of AMPs by the fat body, an insect immune-response tissue, and occasionally by hemocytes and other tissues [68-71]. These peptides are secreted into the hemolymph, where they exhibit a broad range of activities against fungi and bacteria. The mechanisms of AMP action are poorly understood, but at least in some cases (for example, drosomycin in *Drosophila*), AMPs destroy invading microbes by disrupting microbial cell membranes, leading to cell lysis [71].

Antimicrobial peptides are diverse and ubiquitous. They tend to be small molecules (<30 kDa) specialized at attacking particular microbial classes (that is, Gram-positive bacteria, fungi, and so on) [68,69]. While some antimicrobial peptides are found in only a single insect group (for example, metchnikowin is found only in *Drosophila*), others are widely dispersed across eukaryotes (for example, defensins are present in fungi, plants and animals). Genomics, coupled with proteomics, has revealed that all sequenced insects, and many other insects, have multiple types of antimicrobial peptides (Figure 2). Pea aphids, surprisingly, are missing many of the antimicrobial peptides common to other insects. For example, while all insect genomes annotated thus far have genes encoding defensins [13], homology-based searches, phylogenetic-based analyses, transcriptomics (see below), and proteomics (see below) failed to find any signatures of defensins in the pea aphid genome. The presence of defensins in the human louse *Pediculus humanus *(Figure 2), and in the ancient apterygote insect, the fire brat *Thermobia domestica *[34], suggests that defensins have been lost during aphid evolution.

Extensive searches for genes encoding insect cecropins, drosocin (and other proline-rich arthropod AMPs), diptericin (and other glycine-rich AMPs), drosomycin, metchnikowin, formicin, moricin, spingerin, gomesin, tachyplesin, polyphemusin, andropin, gambicin, and virescein also revealed no hits. Weak hits were found for genes that encode for two antimicrobial peptides in other invertebrates: megourin [UniProtKB: P83417], originally isolated from another aphid species, the vetch aphid *Megoura viciae *(P Bulet *et al.*, unpublished data) and penaeidin [UniProtKB: P81058], originally isolated from the shrimp *Penaeus vannamei*. The putative pea aphid *Megourin *(scaffold EQ11086, positions 45,752 to 45,892), however, is highly diverged from that of *M. viciae *(31% identity) and, compared to its *M. viciae *counterparts, seems to have a shorter carboxy-terminal region containing a stop-codon (Figure S2 in Additional file 1). Using three different primer pairs, we were unable to amplify products of this putative *Megourin *from cDNA generated for expression analyses (see below). The highly divergent *Penaeidin *[GenBank: ACYPI37769] (Figure S2 in Additional file 1) also did not amplify from cDNA.

**Figure 3:**
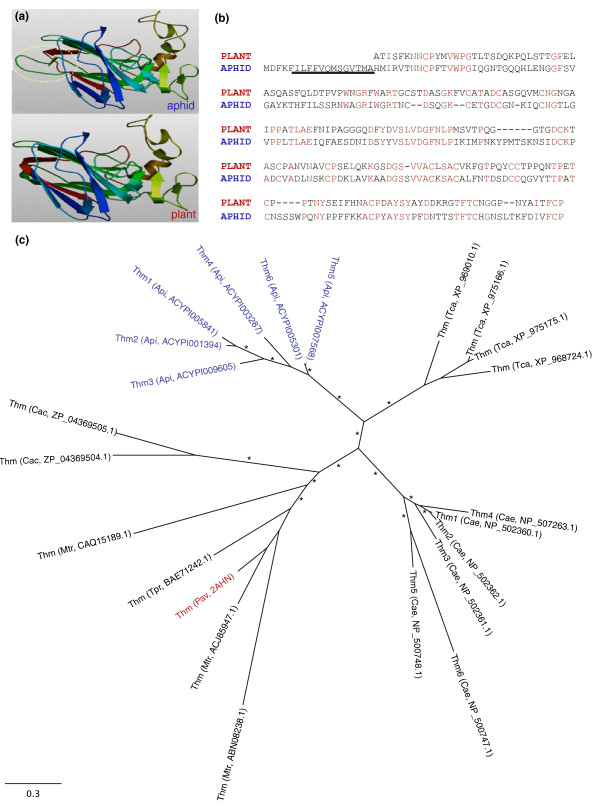
**Evolutionarily conserved thaumatins are present in pea aphids and plants**. **(a) **The three-dimensional structure of the pea aphid thaumatin ACYPI009605 (top) was calculated using the published crystallographic structure of a sweet cherry (plant) thaumatin 2AHN_A (bottom) [130] and Swissmodel [131], revealing that both thaumatins are similar in structure. However, one exposed loop, encircled by a dotted line, shows a significant difference in structure, suggesting possible adaptation to different targets. **(b) **Similarities are also revealed in the alignment of the pea aphid thaumatin with the plant thaumatin. A predicted signal sequence of the pea aphid thaumatin is underlined. Identical amino acids are highlighted in red. **(c) **Maximum likelihood phylogeny of thaumatins, indicating branches leading to nematode, plant, insect and bacteria-specific clades. Red highlights the sweet cherry thaumatin. Blue highlights the pea aphid thaumatins. Asterisks indicate approximate likelihood ratio test support >80. Abbreviations: Api, *A. pisum*; Cac, *Catenulispora acidiphila*; Cel, *Caenorhabditis elegans*; Mtr, *Medicago truncatula*; Pav, *Prunus avium*; Tca, *Tribolium castaneum*; Tpr, *Trifolium pretense*.

We found six *Thaumatin *homologs in the *A. pisum *genome that show overall sequence and predicted structure similarities to plant thaumatins (Figure 3a, b). Thaumatin-like proteins are disulfide-bridged polypeptides of about 200 residues. Some thaumatins possess antifungal activity in plant tissues after infection [72]. Recently, a thaumatin found in the beetle *T. castaneum *was shown to inhibit spore germination of the filamentous fungi *Beauveria bassiana *and *Fusarium culmorum *[32]. Phylogenetic analyses revealed that *A. pisum *thaumatins form a monophyletic group closely related to beetle thaumatins (Figure 3c). Since thaumatin-like genes are conspicuously absent from the genomes of *Drosophila*, *Apis*, *Anopheles*, *Pediculus *and *Ixodes *(Figure 2), our findings indicate that thaumatins may represent ancient defense molecules that have been lost in several insect species, or have been independently acquired in aphids and beetles. The monophyly of aphid and beetle thaumatins provides no indication of an origin of novel acquisition (Figure 3c).

#### Lysozymes

Lysozymes represent a family of enzymes that degrade bacterial cell walls by hydrolyzing the 1,4-beta-linkages between *N*-acetyl-D-glucosamine and *N*-acetylmuramic acid in peptidoglycan heteropolymers [73]. They are ubiquitously distributed among living organisms and are believed to be essential for defense against bacterial infection. Lysozymes are classified into several types (that is, c (chicken), g (goose), i (invertebrate), plant, bacteria and phage types). C-type lysozymes are the most common for metazoa, being found in all vertebrates examined thus far and many invertebrates, including all the previously sequenced insects. For example, *D. melanogaster *and *A. gambiae *have at least seven and nine loci for c-type lysozymes, respectively [74,75]. Insects also have i-type homologs, but their bacteriolytic activities are unclear [76].

Unlike other insects sequenced thus far, similarity searches demonstrated that *A. pisum *lacks genes for c-type lysozymes. The analysis further verified that the genome also lacks genes for g-type, plant-type, and phage-type lysozymes. Only three genes for i-type homologs were detected in the genome (Figure S1d in Additional file 1). One of them, *Lys1*, is highly expressed in the bacteriocyte [77]. Two others, *Lys2 *and *Lys3*, are located adjacent to *Lys1*.

Notably, two genes that appear to have been transferred from bacterial genomes to the *A. pisum *genome encode bacteriolytic enzymes [36]. One is for a chimeric protein that consists of a eukaryotic carboxypeptidase and a bacterial lysozyme. The other (*AmiD*) encodes *N*-acetylmuramoyl-L-alanine amidase, which is not a true lysozyme (1,4-beta-N-acetylmuramidase) but similarly degrades bacterial cell walls. While some of these bacteriolytic-related genes are highly expressed in the bacteriocyte, and lysozymes appear to be upregulated in response to some challenges (see gene expression study, below), assays of bacterioltyic activity of hemolymph from immune-challenged aphids suggest that aphid hemolymph has weak to no lysozyme-like activity [31]. Further studies will determine the role of these gene products.

#### Chitinases

Chitinases are enzymes that degrade chitin (a long-chain polymer of *N*-acetyl-D-glucosamine), hydrolyzing 1,4-beta-linkages between *N*-acetyl-D-glucosamines. Chitinases and lysozymes represent a superfamily of hydrolases, and their catalytic activities are similar. Indeed, some chitinases show lysozyme activity and vice versa [73]. In insects, chitinases are used to degrade the chitin in the exoskeleton and peritrophic membrane during molting, and some are suspected to have antifungal activity, as fungal cell walls also consist of chitin [78]. Similarity searches followed by phylogenetic analyses demonstrated that the genome of *A. pisum *encodes seven genes for putative chitinase-like proteins [79]. Further studies are required to determine the biochemical properties and substrate specificity of these chitinase-like proteins.

#### TEPs and Tots

Some TEPs can covalently attach to pathogens and parasites in order to 'mark' them for phagocytosis [80]. Like other insects, aphids have multiple *Tep *paralogs. Both are homologous to *TepIII *(Figure S1e in Additional file 1). Homologs of *TepI*, *TepII *and *TepIV *were not found. In contrast, no *Turandot *(*Tot*) genes, which encode small peptides induced by severe stress and septic injury in *Drosophila *[81-83], have been found in aphids or in other insects other than *Drosophila *spp. Both TEPs and Tots are thought to be regulated by the JAK/STAT pathway.

#### Prophenoloxidase

Phenoloxidase-mediated melanin formation characteristically accompanies wound clotting, phagocytosis and encapsulation of pathogens and parasites [84]. In insects, the inactive enzyme prophenoloxidase (ProPO) is activated by serine proteases to yield phenoloxidase [85]. Aphids appear to have two prophenoloxidase homologs (*ProPO1*, *ProPO2*; Figure S1f in Additional file 1), which are homologous to *D. melanogaster Diphenol oxidase A3 *[Flybase: CG2952].

#### Nitric oxide synthase

Production of nitric oxide is mediated by the enzyme nitric oxide synthase. Nitric oxide is a highly unstable free radical gas that has been shown to be toxic to both parasites and pathogens. In insects, *Nos *is upregulated after both parasite and Gram-negative bacterial infection [86,87]. Like other insects, pea aphids have one *Nos *homolog.

#### Heat shock proteins

Though called HSPs, these proteins are produced in response to a range of stresses in both eukaryotic and prokaryotic organisms [18]. They serve as chaperones, facilitating protein folding and stabilization, and as proteases, mediating the degradation of damaged proteins. HSPs may also serve as signaling proteins during immune responses [18,88]. In many insects, including aphids, *HSP*s have been shown to be upregulated after septic injury and microbial infection [31,89-92]. We identified 15 *HSP*s of varying molecular weight in pea aphids (Figure S1g in Additional file 1).

#### Gluthione-S-tranferases

Gluthione-S-tranferases comprise a diverse class of enzymes that detoxify stress-causing agents, including toxic oxygen free radical species. They are upregulated in some arthropods upon oxidative stress [93] and microbial challenge [89,94]. Pea aphids have at least 18 genes encoding gluthione-S-tranferases and many other detoxification enzymes that likely play a role in stress responses [95]. Ramsey *et al. *[95] identified many of the genes encoding detoxification enzymes in *A. pisum *and in *Myzus persicae*.

#### Alarm pheromone production

In response to predators, aphids release an alarm pheromone that causes neighboring aphids to become more mobile and to produce more winged than unwinged offspring [19,96]. These winged offspring have the ability to disperse to enemy-free space. While many insects produce a suite of chemicals that constitute an alarm signal, the aphid alarm pheromone is dominated by a single compound, (*E*)-β farnesene [97]. While the genes underlying alarm pheromone production have not been fully characterized, we have identified a *Farnesyl diphosphate synthase *(*FPPS*) and an *Isoprenyl diphosphate synthase *(*IPPS*), which may underlie alarm pheromone production [98].

### Functional assays

#### Gene expression

We utilized real-time quantitative PCR to conduct a preliminary investigation of the expression of 23 recognition, signaling and response genes in aphids subjected to a number of infection and stress treatments (see Supplementary materials and Table S2 in Additional file 1). While future studies with more biological replicates will be necessary to fully survey gene regulation in the face of stress and infection, this initial survey indicates that aphids do express these genes under both control and infection/stress conditions (Tables S4 and S5 in Additional file 1). This suggests that these genes are functional even in the absence of many other missing immune-related genes.

One expression pattern seen in this initial survey is of particular note. Unlike other insect immune expression studies, we found no strong upregulation of antimicrobial peptides, which frequently exhibit ten-fold or greater upregulation in the face of infection. For example, while Altincicek *et al. *[32] observed 20-fold upregulation of *Thaumatins *in tribolium beetles after stabbing with lipopolysaccaride endotoxin derived from *Escherichia coli*, we saw modest upregulation (approximately 2-fold) of only one *Thaumatin *(*Thm2*) after stabbing aphids (Table S5 in Additional file 1). Furthermore, despite the fact that they are known to suppress fungal germination in beetles, the *Thaumatin *homologs were not upregulated after fungal infection at the time point included in this study, and were only approximately two-fold upregulated at two additional time points and in a follow-up fungal infection experiment (data not shown) [32]. The role of thaumatins in fighting microbial infections, however, should not be discounted, as they may function in the absence of significant upregulation (that is, they may be constitutively expressed).

#### Exploration of ESTs from infected and uninfected aphids

In the first of two EST-based experiments, we compared a cDNA library synthesized from the guts of *A. pisum* that had been fed a Gram-negative pathogen, *Dickeya dadantii*[99], to a cDNA library synthesized from uninfected guts. Strikingly, no standard immune-related genes, such as antimicrobial peptides, were identified in the infected sample. The main functional classes differentially expressed were the 'biopolymer metabolism' class, many members of which were down-regulated in infected guts, and 'transport' or 'establishment of localization' classes, whose genes were upregulated in infected guts (Table S6 in Additional file 1). The 'immune response' class, in contrast, was only represented by five genes. Four of these five genes were in the uninfected library, while only one, encoding a leucyl-aminopeptidase, was identified from the infected library; the immune function of leucyl-aminopeptidases is not well understood. Moreover, the 'response to stress/external stimulus/biotic stimulus' classes were not overrepresented in the infected gut library.

In a separate experiment, to further identify aphid immune-relevant genes, we utilized SSH to compare cDNA from *E. coli*-infected aphids and cDNA from unchallenged aphids. To obtain genes expressed at different phases of the immune response, three RNA samples were extracted 3, 6 and 12 hours after *E. coli *infection and mixed prior to cDNA synthesis.

Among the 480 ESTs that were sequenced from the subtracted library [GenBank: GD185911 to GD186390], we found some genes with similarity to proteases and protease inhibitors but few other immune-related proteins. Interestingly, SSH-based EST analysis failed to identify any PRRs, such as PGRPs or GNBPs, or any antimicrobial peptides (Table S7 in Additional file 1). It is noteworthy that this aphid experiment was conducted in parallel to a similar *Sitophilus *weevil experiment, where many immune-related genes (more than 18% of ESTs) were identified, including antibacterial peptides and PRRs [35]. This suggests that the paucity of immune genes identified in *A. pisum *is not a technical issue but may be a specific feature of aphids [31]. In addition, dot blot analysis demonstrated that only a few genes (less than 5%) were differentially expressed between *E. coli*-stabbed and unstabbed aphids. These findings indicate that, in contrast to other insects, either aphids respond only weakly to challenge with *E. coli *or aphid genes and pathways directed against these bacteria are expressed only constitutively.

#### High performance liquid chromatography

**Figure 4:**
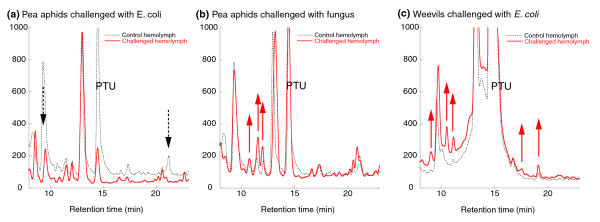
**HPLC traces of inducible hemolymph peptides in the pea aphid compared to the rice weevil**. Representative traces (solid, red lines) are from insects 18 hours after microbial challenge; traces generated from 18 hour control insects are overlaid (dashed, black lines). Phenylthiourea (PTU) served as an internal standard. Arrows indicate peaks that are significantly upregulated (solid, red arrows) or downregulated (dashed, black arrows). **(a) **Profile from pea aphids challenged with *E. coli*, showing no upregulated response. **(b) **Profile from pea aphids challenged with the fungus *A. fumigatus*, showing some differential peaks. **(c) **For comparison, profile from rice weevils (*Sitophilus oryzae*) challenged with *E. coli*, showing several differentials peaks at multiple retention times.

HPLC peptide analyses targeting production of small peptides (for example, antimicrobial peptides) were run on hemolymph samples from pea aphids challenged by three microorganisms: *E. coli *(Gram-negative bacteria), *Micrococcus luteus *(Gram-positive bacteria) and *Aspergillus fumigatus *(fungi). Profiles were compared between control, infected and sterile-stabbed aphids at 6, 12 and 18 hours after challenge. When identified, the production of small peptides was maximal at 18 hours. In *E. coli*-treated samples, no upregulation could be identified (Figure 4a), in *M. luteus*-treated samples, there was modest upregulation (data not shown), and in *A. fumigatus*-treated samples, there was a significant response, though few peaks (Figure 4b). In contrast, a response profile to *E. coli *from another obligate symbiotic insect (the weevil, *Sitophilus oryzae*) exhibited at least five well-distinguishable upregulated peaks (Figure 4c). Response being restricted to Gram-positive bacteria and fungi is consistent with previous identification of megourin, an antimicrobial peptide in the aphid *Megoura viciae*, which appears to have activity against Gram-positive bacteria and fungi, but not against Gram-negative bacteria (P Bulet, unpublished). Because so few distinguishable peaks were present in the aphid samples, we did not choose to identify the associated products, but overall the presence of few inducible peptides suggests a peculiar scarcity of antimicrobial peptides in aphids.

## Conclusions

Aphids are one of only a few genomic models for hemimetabolous insects, yet until recently, virtually nothing was known about aphid immune and stress response systems. Here, by coupling gene annotation with functional assays, we see evidence that aphids have some defense systems common to other arthropods (for example, the Toll and JAK/STAT signaling pathways, HSPs, ProPO). Surprisingly, however, several of the genes thought central to arthropod innate immunity are missing in aphids (for example, PGRPs, the IMD signaling pathway, defensins, c-type lysozymes). This calls into question the generality of the current model of insect immunity, and it remains to be determined how aphids protect themselves from the diverse pathogens and parasites that they face.

The fact that we cannot find aphid homologs to many insect immune genes could be a consequence of the large evolutionary distance between aphids and the taxa (in most cases, flies, mosquitoes and bees) from which these genes are known (that is, the split between the ancestors of aphids and these taxa occurred approximately 350 million years ago [100]), making it challenging to find divergent genes via homology-based searches, even when using highly sensitive methods as done here. Though we cannot preclude this possibility in all cases, in some cases, similar homology-based methods are able to recover homologs in even more distantly related taxa. For example, querying genome databases with *Drosophila *genes via BLAST recovers putative homologs of PGRPs and defensins in *P. humanus *(human body louse) and in *Ixodes scapularis *(deer tick) (Figure 2). The divergence time between *Drosophila *and these taxa is equal to or greater than that between *Drosophila *and aphids. Moreover, for some cases, we could identify genomic regions similar to functional genes in other species, but these regions contain large insertions or stop codons (for example, the putative antimicrobial peptide Megourin), indicating they are the result of pseudogenization.

One potential explanation for the lack of known immune-related genes in pea aphids is that aphids mount an alternative, but equal, immune response. Our functional analyses, as well as those of Altincicek *et al. *[31], found little evidence for an alternative response. In EST and HPLC analyses, few novel ESTs or peptide signals were recovered from immune-challenge aphids relative to their unchallenged controls. It should be noted, however, that these challenges were primarily limited to exposure to *E. coli *bacteria. When testing for expression of a few immune genes in response to a wider array of challenges, we do see some evidence of an aphid immune and stress response. Future expression studies, including large-scale transcriptional and proteomic studies, will extend this work and allow for more comprehensive characterization of the full complementation of aphid immune responses.

While we have focused mainly on the humoral component of the innate immune response, it is interesting to note that there is some evidence that the cellular component of pea aphids' innate immune response may also be different to that seen in other insects. While many insects encapsulate parasitoid wasp larvae, smothering them to death with hemocytes (insect immune cells), aphids appear not to have this layer of protection [101,102]. Aphids, however, appear to recruit some hemocytes to parasitoid eggs, suggesting that cellular immunity may play an alternative, though possibly more limited, role [101]. Better insights into the capacity of the aphid immune system will require further investigation of both the humoral and cellular components of aphid immunity.

The lack of genomic and molecular data regarding immune systems of aphid relatives makes it difficult to establish whether the pea aphid immune system is unique. There are, however, a number of aspects of aphid ecology that could facilitate ecological success without a strong immune defense. Altincicek *et al. *[31] proposed three hypotheses to explain the apparent lack of antimicrobial defenses. First, they suggested that contrary to *Drosophila*, whose natural environment consists of decaying fruit that is colonized by many microbes, aphids exploit phloem sap, which only occasionally contains bacteria and rarely contains entomopathogens. Thus, the risk of encountering pathogens while feeding is more limited. This assumption, however, is only partly true. While probing plants, aphids are capable of acquiring pathogenic bacteria from the surface of their host plants' leaves [103], and aphids become host to a diverse assemblage of bacteria and fungi under stressful conditions [104], some of which are pathogenic (NM Gerardo, unpublished data). Furthermore, *Sitophilus *weevils, which when challenged with *E. coli *significantly upregulate immune genes [35], spend their entire larval and nymph stages within sterile cereal grains, indicating that a sterile diet is not likely to explain the absence of antibacterial defenses in aphids.

Altincicek *et al. *[31] also suggest that aphids may invest in terminal reproduction in response to an immune challenge, rather than in a costly immune response. In their study, stabbed aphids produced significantly more offspring than untreated aphids within 24 hours of injury. Such an increase in reproduction upon challenge is not uncommon for invertebrates. *Biomphalaria *snails [105,106], *Acheta *crickets [107], *Daphnia *waterfleas [108], and *Drosophila *flies [109] have all been shown to increase their investment in reproduction in response to infection. Yet, *Drosophila *still mount a complex immune response. Furthermore, aphids do not increase their reproductive effort in the face of all immune challenges: fungal infection reduces the number of offspring *A. pisum *produce within 24 hours of inoculation [110], and response to stabbing with bacteria seems to be specific to the aphid genotype and to the location of the stab (Barribeau, unpublished data). Therefore, though aphids have the capacity to reproduce many offspring prior to succumbing to some pathogens, it seems that immune competence would still provide increased fitness.

Even without increased reproduction following infection, the prolific reproductive capacity of aphids suggests these insects, in general, may invest most resources towards rapid, early onset reproduction rather than towards fewer, though better-protected offspring (aka, in terms of classical ecological theory, aphids may be r-selected rather k-selection organisms [111]). Recent theory of the evolution of immunity suggests that such organisms may specifically invest less in costly immune responses [112,113]. Many characteristics of aphids, including their rapid generation time, short life span and small body size all fit a model of r-selection [114]. *Drosophila *spp., however, also exhibit many of these characteristics and still invest in a strong defense repertoire.

The third hypothesis proposed by Altincicek *et al. *[31] concerning the evolution and maintenance of aphid defense relies on the presence of secondary symbionts that can be found extracellularly in aphids [115]. *A. pisum *is protected against fungal pathogens by one of these secondary symbionts, *Regiella insecticola *[29], and also against the parasitoid wasp *Aphidius ervi *by another secondary symbiont, *Hamiltonella defensa *[27]. Such symbiont-mediated host protection may explain why aphids have a reduced (or specialized) antimicrobial defense. This hypothesis seems plausible with regard to the cost of immune gene expression versus the benefit of protection by the secondary endosymbionts. However, it does not explain how the secondary endosymbionts (as Gram-negative bacteria), often present in aphid hemolymph, are themselves perceived and controlled by the aphid immune system. Thus, it is challenging to say whether the presence of secondary symbionts is a cause or a consequence of reduced antimicrobial activity.

Potentially, all of these forces could shape the evolution of aphid stress and immune responses. In order to test these hypotheses (for example, reproductive investment, symbiont-mediated host protection), we need more studies characterizing the global aphid response under more conditions, and in more aphid species. Potential insight from aphid relatives with different lifestyles (for example, those not associated with secondary symbionts, or those that live in soil or other microbe-rich habitats) may be particularly helpful. More broadly, as the pea aphid is the first published genome of a hemimetabolous insect, future analyses of the immune and stress related genes of more insects in this group will facilitate the reconstruction of the evolutionary history of innate immunity and other defenses.

## Materials and methods

### Bioinformatic screening of the pea aphid genome

Immune and stress gene candidates from other insects (for example, *D. melanogaster*, *A. aegypti*, *A. gambiae*, *A. mellifera*) were used to query the pea aphid genome. Most searches utilized the blastp search function to search for hits against the predicted *A. pisum *proteome [116]. For some gene families and putative paralogs, protein sequences were aligned to sequences from other insects and outgroups using ClustalW [117]. These alignments, as well as available EST and full length cDNA sequences, served to refine aphid gene models (exon/intron boundaries, and so on), and to facilitate phylogenetic analyses. In addition, a comprehensive database of all available EST sequences from the green peach aphid, *Myzus persicae*, was screened using tblastn to search for potential homologs to all immune and stress genes annotated in the pea aphid.

For genes that could not be found in the proteome, we also conducted a tblastn search against all contigs and unassembled reads. Then, a final, more sensitive profile-based search was performed for those immune defense proteins that produced no hits with BLAST searches. For this analysis, insect and other species protein sequences belonging to the family of interest were retrieved from NCBI and aligned with MUSCLE [118]. A hidden Markov model for the alignment was built and calibrated using HMMER [119]. This was used to perform a profile-based search (hmmsearch) against the six-frame translated sequences of the assembled pea aphid genome and the unassembled reads. Additionally, a similar search with PFAM profiles [120] was also performed for those families encoding PFAM domains in their sequences. Whenever a significant hit was found, the genomic region was analyzed to discard the possibility that it encoded a pseudogene (presence of stop codons, absence of relevant domains, and so on).

Phylogenetic analyses of selected protein families were performed using their corresponding maximum likelihood phylogenetic trees from the pea aphid phylome [36], deposited in PhylomeDB [121]. When necessary, additional sequences were added to the original PhylomeDB alignment, realigned with MUSCLE and used to reconstruct a maximum likelihood phylogenetic tree, using the JTT (Jones-Taylor-Thornton) model as implemented in PhyML v2.4.4 [122], assuming a discrete gamma-distribution model with four rate categories and invariant sites, and estimating the gamma shape parameter and the fraction of invariant sites. Cladograms were edited using Dendrogram [123].

### Exploration of ESTs from infected and uninfected aphids

In the first experiment, two EST libraries (one control, one infected) were generated by standard procedures using a SMART cDNA kit (Clontech, Mountain View, California, USA), starting from approximately 1,000 dissected *A. pisum *midguts for each library. The aphids were clonal, young, reproducing asexuals, which were either fed on control diet or infected by feeding on artificial diet with the Gram-negative aphid pathogen *Dickeya dadantii *at 10^6 ^bacteria per milliliter [99]. Twenty-four hours after infection, control and treated aphids were dissected, and complete guts were transferred immediately into RNeasy solution (Qiagen Valencia, California, USA). ESTs were sequenced according to procedures in Sabater-Munoz *et al. *[124].

In another EST-based experiment utilizing SSH and dot-blot technology, we treated aphids (clone LL01) with rifampicin as described in Rahbé *et al. *[125] to reduce symbiont load. We challenged wingless fourth-instar aposymbiotic aphids by stabbing them with needles previously dipped into a pellet of overnight cultures of *E. coli *(TOP10, Invitrogen Carlsbad, California, USA), and then maintained them on fava plants. At 3, 6, and 12 hours post-treatment, we stored surviving aphids at -80°C. To identify genes that are differentially expressed in response to septic injury, we performed SSH using RNAs from immune challenged (3, 6 and 12 hours post-treatment) and untreated aposymbiotic aphids, using the SMART PCR cDNA Synthesis Kit and the PCR-Select cDNA Subtraction Kit (Clontech) according to the manufacturer's instructions and as described in Anselme *et al. *[35]. After transformation by electroporation, we recovered approximately 1,500 colonies from LB agar plates. We plasmid extracted and sequenced 500 randomly picked colonies (NucleoSpin^® ^Plasmid Kit, Macherey-Nagel, Düren, Germany) utilizing the sequencing center at the University of Valencia (Spain). We compared all sequences against UniProt using blastx. Immune-related gene sequences (Table S7 in Additional file 1) were then compared to the aphid genome using blastn.

To analyze the differential expression status of each EST, we conducted a dot-blot experiment. Briefly, we amplified 344 ESTs from the SSH library by colony PCR with nested PCR primers 1 and 2R from the PCR-Select cDNA Subtraction Kit. We then spotted 10 μl from each PCR product onto two different membranes (Amersham Hybond™-N, GE Healthcare Life Sciences, Piscataway, New Jersey, USA) using a *Bio-Dot *Microfiltration System (Biorad, Hercules, California, USA). We hybridized membranes with radiolabeled cDNA probes generated by reverse-transcription from RNA extracted from either aposymbiotic aphids stabbed with *E. coli *or unstabbed aposymbiotic aphids. We synthesized these probes using the Super Script™ First Strand Synthesis system (Invitrogen) for RT-PCR and [α-^32^P]dCTP, and purified them using *Quick Spin *Column (Roche Molecular Biochemicals, Indianapolis, Indiana, USA). After exposing blots for up to 24 hours to a Storm PhosphorImager imaging plate (GE Healthcare Life Sciences), we analyzed differential expression by comparison of band intensities between the two membranes. We did not, however, normalize the data, as we failed to see any signal from the *Gapdh *gene, though the same amount of each PCR product was loaded on both membranes.

### HPLC

Aphids were challenged by abdominal puncture with triple-0 needles dipped in a solution of Gram-negative bacteria (*E. coli *strain Top10), Gram-positive bacteria (*M. luteus*) or fungal spores (*A. fumigatus*). For each microbial treatment, five hemolymph samples from 50 aphids each were collected at four times points (t = 0, 6, 12 and 18 hours).

Hemolymph was flash-extracted by centrifuging (1 minute, 10,000 g, 4°C) live aphids through a 1 ml pipette tip and directly into 40 μl 0.1% trifluoractetic acid contaning 10 μl of saturated phenylthiourea (PTU) for phenoloxidase inhibition. Resulting samples were highly similar to pure hemolymph samples obtained by leg bleeding (>95% band identity by silver-stained SDS-PAGE).

After initial collection, tips were removed and the samples were centrifuged for 5 minutes at 15,000 g. Following addition of 70 μl trifluoractetic acid 0.1%, the supernatant sat for 1 hour at 4°C to allow for protein precipitation prior to a final 10-minute centrifugation at 15,000 g to recover peptides. Samples were evaporated and stored at -20°C until use in HPLC. Chromatography was performed on standard peptide C18-300Å reverse phase columns using water acetonitrile gradients [126]. For retention time standardization, PTU served as an internal standard, and samples were analyzed by area-normalization to unchallenged sample peaks (retention time = 14 minutes, preceding PTU).

## Abbreviations

AMP: antimicrobial peptide; EST: expressed sequence tag; GNBP: Gram-negative binding protein; HPLC: high performance liquid chromatography; HSP: heat shock protein; IMD: immunodeficiency; JAK/STAT: Janus kinase/Signal transducers and activators of transcription; JNK: c-Jun N-terminal kinase; PGRP: petidoglycan receptor protein; ProPO: prophenoloxidase; PTU: phenylthiourea; PRR: pathogen recognition receptor; SSH: suppression subtractive hybridization; TEP: thiolester-containing protein.

## Authors' contributions

NMG, SMB, and MG were group leaders for the project. NMG, BA, HA, SMB, MDV, EJD, JDE, AM, MG, IK, AN, BJP, MP, JSR, JT, DT, and CT designed and performed manual gene annotation. TG and SMB conducted phylogenetic analyses. BA and AV conceived of and conducted analyses of Thaumatin. SMB, NMG, CS and BJP performed experiments and analyses for the gene expression study. CA, AH, VPB, AM, and AL conceived of and conducted the SSH study, and CVM constructed the aphid gut libraries. YR conducted the HPLC study. The manuscript was prepared by NMG, SMB, CA, TG and YR with input from MDV, BA, AN, AV and AH. All authors have read and approved the final version of the manuscript.

## Supplementary Material

Additional file 1 Table S1: pea aphid immune and stress gene list. Table S2: samples for quantitative PCR expression study. Table S3: primers for quantitative PCR expression study. Table S4: relative expression of recognition and signaling genes. Table S5: relative expression of response genes. Table S6: gut EST library statistics. Table S7: list of selected ESTs from the subtracted library. Figure S1: maximum likelihood phylogenies of selected immune and stress gene families. Figure S2: alignments of putative antimicrobial peptides megourin and penaeidin. Figure S3: survival curves for experimental infections associated with quantitative PCR study. Click here for file
