# Supplementary material for: Immunity and other defenses in pea aphids, Acyrthosiphon pisum
Source: Genome Biol. 2010 Feb 23;11(2):R21. doi: 10.1186/gb-2010-11-2-r21 (PMC2872881; doi:10.1186/gb-2010-11-2-r21)

Additional file 1

Supplemental Material: Immunity and defense in pea aphids, *Acyrthosiphon pisum*

Supplementary Methods for Gene Expression Study

Gene expression overview. We utilized real-time quantitative PCR to conduct a preliminary investigation of the expression of 23 recognition, signaling and response genes in aphids subjected to a number of infection and stress treatments (Tables S2, below). First, we verified expression of the immune and stress genes in aphids stabbed with needles inoculated with the natural commensal *Escherichia coli*, a natural Gram-positive bacterial pathogen, a natural Gram-negative bacterial pathogen, or with no needle (control). More closely mimicking the uptake of bacteria while feeding, we verified expression of the genes in aphids fed on artificial diet containing either *E. coli* or a Gram-negative pathogen relative to that of aphids fed on control diet with no bacteria. We also verified gene expression in entomopathogen fungus-infected to uninfected aphids, and in aphids stabbed with pathogenic virus-inoculated needles to unstabbed control aphids. All microbes used have been isolated from pea aphids in natural populations. Finally, to assess effects of environmental stress, we verified gene expression in aphids exposed to alarm pheromones, starvation, and heat-shock.

We conducted the five challenge experiments with aphids of clonal line LSR1, the clone sequenced in the genome sequencing project. This line was cured of its secondary symbiont, *R. insecticola*, more than two years ago using a standard antibiotic treatment [132], and has been continuously maintained as an asexual clone on fava beans at 16hr

light: 8hr dark at 20°C. For each experiment, we used 5-6 day old, unwinged aphids, which were maintained on fava bean plants at 20°C unless otherwise noted.

Bacterial stabbing experiment. For the first experiment, we stabbed aphids with *E. coli*, Gram-negative pathogen Ng5B, in the genus *Enterobacter*, or Gram-positive pathogen 6B, in the genus *Staphylococcus*. *E. coli* has been previously shown to be a commensal that has little affect on aphid survival when either injected into or fed to aphids (Figure S3, below) [133]. Ng5b and 6b are bacteria originally isolated from laboratory pea aphids. When fed to aphids, these pathogens kill most aphids in approximately 48 hours (Figure S3a, below). The night before the infection, we grew bacterial cultures from glycerol stocks on Luria broth (LB) agar at 37°C. The morning of the infection, we transferred bacterial colonies to LB and grew them at 37°C. We determined concentration of broth cultures by optical density (OD_{600}), and then standardized the cultures to $OD_{600} = 0.5$. Next, we stabbed aphids with a minutin pin contaminated with the standardized bacterial cultures or with sterile LB and then transferred the stabbed aphids and unstabbed control aphids to fava bean plants. After eight hours, we froze five aphids per condition in liquid nitrogen and stored them at -80°C for subsequent RNA extraction. We monitored ten aphids per condition for survival (Figure S3, below).

Bacterial feeding experiment. For the second experiment, we fed aphids on AP3 artificial *A. pisum* diet [134] containing no bacteria (control), *E. coli*, or the Gram-negative pathogen Ng5b. We grew bacteria as above and then inoculated the treatment diets with 1 μL bacterial culture per 20 mL diet, and control diet with the same amount of bacteria-free LB. We plated the diets onto LB agar to confirm that the control diet was

free of bacteria and that the final bacterial concentration in the treatment diet was approximately 2 x 10^4 colony forming units (cfu) per mL. We added one drop of food-grade blue dye per 5 mL of diet to allow us to detect whether aphids had ingested the diet. We then filled 10 mm Petri dish bottoms with diet (either control or treatment) and covered the dish with stretched Parafilm. We affixed the feeding dishes to the bottom of 15 mm Petri dishes, and transferred 30 - 50 aphids directly from plants to each feeding dish. We maintained dishes upside down in a 20°C incubator. Approximately 12 hours after being exposed to the diet, we froze five aphids from each treatment that had fed (as determined by the presence of dye in the aphid intestinal tract) in liquid nitrogen and maintained them at -80°C for subsequent RNA extraction. Eight hours later, we transferred the remaining aphids that had fed (n = 30 per treatment) to fava bean plants and monitored them for survival (Figure S3a, below).

Fungal shower experiment. For the third experiment, we exposed aphids to a shower of spores of the fungus *Zoophthora occidentalis,* an aphid specific fungal entomopathogen [135]. We placed aphids in a 70 mm tall cylinder with a damp sponge at the bottom. We then inverted an approximately two-week-old culture of *Z. occidentalis* on potato dextrose agar (PDA) over the cylinder and allowed the spores to fall on the aphids for 2 hours. Control aphids were exposed to the same conditions but with a sterile PDA plate. After exposure, we transferred the aphids to fava plants. We froze five aphids per condition in liquid nitrogen 24 hours after exposure and monitored 22 aphids per condition for survival (Figure S3b, below). The frozen samples were maintained at -80°C for subsequent RNA extraction.

Viral stabbing experiment. For the fourth experiment, we stabbed aphids with a solution containing Aphid Lethal Paralysis Virus (ALPV), a RNA virus found in natural populations that is lethal to pea aphids (Georgievska, Miller & Bonning, unpublished data) [136, 137]. The solution was made by grinding up virus-killed aphid cadavers in 7.5 μL of water per cadaver. After stabbing, aphids were isolated for an hour and then kept on fava bean plants until 16 hours from initial stabbing, when five aphids per condition were removed from the plants, frozen in liquid nitrogen, and stored at -80°C prior to RNA extraction. Unstabbed control aphids, raised and treated under similar conditions, were frozen as well. In addition, 20 stabbed and 20 unstabbed aphids were monitored over a period of 96 hours for survival (Figure S3c, below). Though it is possible that responses to stabs with the virus-slurry would be a result of exposure to other pathogens in the ground-up cadaver, stabs with slurry from non-virus killed aphid cadavers do not lead to a significant increase in mortality (Parker, unpublished data), and thus we expect that responses can be largely attributed to virus exposure.

Stress experiment. For the fifth experiment, we exposed aphids to a number of stressors. We exposed one group of aphids on plants to 3 μL of (*E*)-β farnesene (EBF) (1 μg/μL in hexane), which we placed on a small piece of filter paper near the base of each plant. We enclosed the plants under a plastic cover with no ventilation. Within minutes of exposure, we froze five EBF-exposed aphids, and five control, unexposed aphids. Twelve hours later, we froze another five aphids that had been exposed to EBF. We heat shocked a second group of aphids following standard procedures [138]. After two hours at 36°C, we froze five heat-shocked aphids and then maintained another five heat-shocked aphids at 20°C for another eight hours prior to freezing. To assess the affects of starvation, we

placed a third batch of aphids into sterile Petri dishes with moistened filter paper for 12 hours prior to freezing.

Quantitative PCR. For each sample, we extracted RNA from five whole aphids using a Qiagen RNA easy tissue kit and prepared cDNA from each sample using a Qiagen Quantitect reverse transcription kit. We carried out expression studies, utilizing the delta-delta CT method, on an Applied Biosystems Step One Plus machine. Each reaction contained 10 μL AB Power SYBR PCR master mix, 300 nM of each primer, approximately 100 ng cDNA, and water to a total volume of 20 μL. We designed primers utilizing Primer3 [139] or Primer Express (Applied Biosystems) to amplify approximately 100 bps of the gene of interest (Table S3, below). Primers spanned an exon-exon boundary where possible. For each sample, we carried out three separate reactions for each primer pair, and averaged the comparative threshold cycle (Ct) among the three values. We standardized all Ct values for the gene of interest relative to the Ct values for the endogenous control gene actin, yielding the delta Ct value. We then standardized relative to the appropriate control, yielding the delta-delta Ct value. Finally, these delta-delta Ct values were standardized such that each control treatment average was one, yielding the relative quantity (RQ) values, to allow for comparison with studies reporting fold-changes.

Table S1. Pea aphid immune and stress gene list. Genes are listed in the approximate order to which they are mentioned in the text. The last column indicates results of a tblastn search (contig ID and e-value) of all identified pea aphid genes against an EST sequence database for *Myzus persicae*, the green peach aphid. All pea aphid sequences are accessible at AphidBase Gbrowse [140]. All green peach aphid sequences can be downloaded at AphidBase Downloads (files Myzus454 and MyzusSanger) [141].

Class	Role in Insect Immunity	Gene Symbol	Gene Name	Other Gene Names	ACYPI identifier	NCBI gene ID	NCBI transcript ID	NCBI protein ID	*Myzus* ID (e-value)
recognition	bacterial recognition	PGRP	Peptidoglycan recognition protein		not found	not found	not found	not found	n/a
recognition	bacterial and fungal pattern recognition	GNBP1	Gram Negative Binding Protein 1		ACYPI005376	100164352	XM_001944438.1	XP_001944473.1	6372 (7e-134)
recognition	bacterial and fungal pattern recognition	GNBP2	Gram Negative Binding Protein 2		ACYPI006143	100165182	XM_001947795.1	XP_001947830.1	3883 (4e-178)
recognition	bacterial recognition, induction of phenoloxidase	Ctl1	C-type Lectin 1		ACYPI004676	100163601	XM_001944997.1	XP_001945032.1	26603 (3e-39)
recognition	bacterial recognition, induction of phenoloxidase	Ctl2	C-type Lectin 2		ACYPI005998	100165024	XM_001946121.1	XP_001946156.1	26603 (3e-38)
recognition	bacterial recognition, induction of phenoloxidase	Ctl3	C-type Lectin 3		ACYPI004682	100163607	XM_001943575.1	XP_001943610.1	60035 (3e-6)
recognition	bacterial recognition, induction of phenoloxidase	Ctl	C-type Lectin galactose-binding		ACYPI009411	100168734	XM_001951375.1	XP_001951410.1	3644 (3e-130)
recognition	bacterial recognition, induction of phenoloxidase	Ctl	C-type Lectin selectin-like		ACYPI003045	100161853	XM_001942942.1	XP_001942977.1	73637 (1e-31)
recognition	several roles have been hypothesized	gale1	galectin 1	Galactoside-binding soluble lectin	ACYPI001371	100160038	XM_001943734.1	XP_001943769.1	30445 (2e-6)
recognition	several roles have been hypothesized	gale2	galectin 2	Galactoside-binding soluble lectin	ACYPI000409	100158995	XM_001947578.1	XP_001947613.1	24692 (8e-32)
recognition	bind to lipoproteins, bacteria	sr-CI	Scavenger receptor class C, type I		not found	not found	not found	not found	n/a
recognition	bind to lipoproteins, bacteria	sr-CII	Scavenger receptor class C, type II		not found	not found	not found	not found	n/a
recognition	bind to lipoproteins, bacteria	sr-CIII	Scavenger receptor class C, type III		not found	not found	not found	not found	n/a
recognition	bind to lipoproteins, bacteria	sr-CIV	Scavenger receptor class C, type IV		not found	not found	not found	not found	n/a
recognition	receptor in phagocytosis and microbial binding	eater	eater		not found	not found	not found	not found	n/a
recognition	receptor in phagocytosis and microbial binding	nim-C1	nimrod C1		not found	not found	not found	not found	n/a
signaling	toll pathway	spz1-1	spätzle 1Bi	spatzle 1b	ACYPI004362	100163265	XM_001950373.1	XP_001950408.1	649 (1e-99)
signaling	toll pathway	spz1-2	spätzle 1Bii	spatzle 1b	ACYPI001858	100160571	XM_001947931.1	XP_001947966.1	649 (6e-119)
signaling	toll pathway	spz1-3	spätzle 1-3	spatzle 1b, spaetzle 1b	ACYPI41073				649 (4e-83)
signaling	toll pathway	spz1-4	spätzle 1-4	spatzle 1b, spaetzle 1b	ACYPI52992				649 (5e-71)

Class	Role in Insect Immunity	Gene Symbol	Gene Name	Other Gene Names	ACYPI identifier	NCBI gene ID	NCBI transcript ID	NCBI protein ID	*Myzus* ID (e-value)
signaling	toll pathway	spz1-5	spätzle 1-5	spatzle 1b, spaetzle 1b	ACYPI21155				649 (3e-65)
signaling	toll pathway	spz2	spätzle 2	spatzle 2	ACYPI003414	100162252	XM_001948424.1	XP_001948459.1	649 (2e-10)
signaling	toll pathway	spz3	spätzle 3	spatzle 3	ACYPI55738				649 (3e-4)
signaling	toll pathway	spz4	spätzle 4	spatzle 4	ACYPI006811	100165897	XM_001949337.1	XP_001949372.1	no hit
signaling	toll pathway	spz6	spätzle 6	spatzle 6	ACYPI001990	100160712	XM_001944011.1	XP_001944046.1	no hit
signaling	toll pathway	spz-like	spätzle-like, partial	spatzle, spaetzle	ACYPI009165	100168467	XM_001946778.1	XP_001946813.1	no hit
signaling	some tolls function in toll signaling pathway	18w	18 wheeler	Toll, Toll-2	ACYPI008698	100167952	XM_001946908.1	XP_001946943.1	6369 (6e-28)
signaling	some tolls function in toll signaling pathway	Toll	Toll-like		ACYPI000177	100158739	XM_001942698.1	XP_001942733.1	5712 (4e-115)
signaling	some tolls function in toll signaling pathway	Toll	Toll-like		ACYPI002340	100161089	XM_001946411.1	XP_001946446.1	5712 (4e-122)
signaling	some tolls function in toll signaling pathway	Toll	Toll-like, partial		ACYPI004287	100163187	XM_001949147.1	XP_001949182.1	5712 (2e-109)
signaling	some tolls function in toll signaling pathway	Toll-6	Toll-6		ACYPI005417	100164395	XM_001947289.1	XP_001947324.1	6369 (1e-34)
signaling	some tolls function in toll signaling pathway	Toll	Toll-like		ACYPI008268	100167471	XM_001950727.1	XP_001950762.1	6369 (5e-19)
signaling	some tolls function in toll signaling pathway	Tollo	Tollo	Toll-8	ACYPI002754	100161538	XM_001948531.1	XP_001948566.1	6369 (1e-44)
signaling	toll pathway	tub	tube	interleukin-1 receptor-associated kinase 4	ACYPI006580	100165647	XM_001950581.1	XP_001950616.1	4836 (2e-113)
signaling	toll pathway	Myd88	myeloid differentiation primary response gene		ACYPI001638	100160335	XM_001948285.1	XP_001948320.1	1295 (9e-168)
signaling	toll pathway	pll	pelle		ACYPI009928	100169297	XM_001942995.1	XP_001943030.1	4836 (2e-38)
signaling	toll pathway	cact	cactus		ACYPI006820	100165906	XM_001950793.1	XP_001950828.1	259 (3e-135)
signaling	toll pathway	cactin	cactin		ACYPI006968	100166064	XM_001952252.1	XP_001952287.1	2493 (0)
signaling	toll pathway	Pli	Pellino		ACYPI001694	100160395	XM_001946247.1	XP_001946282.1	9336 (3e-71)
signaling	toll pathway	Traf1	TNF-receptor-associated factor 1		ACYPI000855	100159489	XM_001948320.1	XP_001948355.1	24857 (2e-7)
signaling	toll pathway	Traf2	TNF-receptor-associated factor 2	dTraf2, Traf6, TNF-receptor-associated factor 6	not found	not found	not found	not found	n/a
signaling	toll pathway	dl	dorsal		ACYPI003588	100162436	XM_001947394.1	XP_001947429.1	6239 (2e-119)
signaling	toll pathway	dlB	dorsal B		ACYPI005133	100164092	XM_001949463.1	XP_001949498.1	6239 (3e-124)
signaling	jak/stat pathway	dome-1	domeless 1		ACYPI21995	100294629			57862 (9e-8)

Class	Role in Insect Immunity	Gene Symbol	Gene Name	Other Gene Names	ACYPI identifier	NCBI gene ID	NCBI transcript ID	NCBI protein ID	*Myzus* ID (e-value)
signaling	jak/stat pathway	dome-2	domeless 2		ACYPI004970	100163919			57862 (6e-8)
signaling	jak/stat pathway	dome-3	domeless 3		ACYPI21996	100294630			57862 (3e-8)
signaling	jak/stat pathway	dome-4	domeless 4, 5' partial		ACYPI40957	100294721			no hit
signaling	jak/stat pathway	Jak	Janus kinase	hopscotch	ACYPI008118	100167312	XM_001948012.1	XP_001948047.1	1739 (2e-158)
signaling	jak/stat pathway	Stat92E-1	Signal-transducer and activator of transcription 1	Stat	ACYPI002351	100161101	XM_001946610.1	XP_001946645.1	2173 (4e-114)
signaling	jak/stat pathway	Stat92E-2	Signal-transducer and activator of transcription 1 , partial	Stat	ACYPI005642	100164649	XM_001943623.1	XP_001943658.1	65719 (5e-27)
signaling	jak/stat pathway	upd	unpaired		not found	not found	not found	not found	n/a
signaling	imd pathway	imd	immune deficiency		not found	not found	not found	not found	n/a
signaling	imd pathway	dFadd	dFadd	*Drosophila BG4, FADD*	not found	not found	not found	not found	n/a
signaling	imd pathway	Dredd	Death related ced-3	caspase 8	not found	not found	not found	not found	n/a
signaling	imd pathway	Rel	Relish	Nf-KB, REL	not found	not found	not found	not found	n/a
signaling	imd pathway	Tab2	TAK1-associated Binding Protein2		ACYPI002796	100161584	XM_001950344.1	XP_001950379.1	5307 (5e-106)
signaling	imd pathway	Tak1	TGF-β activated kinase 1		ACYPI001063	100159713	XM_001944422.1	XP_001944457.1	2842 (3e-88)
signaling	imd pathway	key	kenny	IKKgamma	not found	not found	not found	not found	not found
signaling	imd pathway	Iap2	Inhibitor of apoptosis 2		ACYPI000445	100159034	XM_001942899.1	XP_001942934.1	60268 (2e-27)
signaling	imd pathway	ird5	immune response deficiency 5	IKK	ACYPI004933	100163880	XM_001952347.1	XP_001952382.1	7578 (3e-90)
signaling	jnk pathway	hep	hemipterous		ACYPI005993	100165019	XM_001944492.1	XP_001944527.1	8122 (8e-46)
signaling	jnk pathway	bsk	basket	MAPK, JNK	ACYPI004372	100163276	XM_001945425.1	XP_001945460.1	1929 (1e-88)
signaling	jnk pathway	Jra	Jun-related antigen		ACYPI002386	100161138	XM_001947521.1	XP_001947556.1	6809 (1e-89)
signaling	jnk pathway	kay	kayak		not found	not found	not found	not found	n/a
signaling	JNK pathway	egr	eiger		ACYPI001133	100159786	XM_001952555.1	XP_001952590.1	1287(2e-131)
response	antimicrobial peptide		Abaecin		not found	not found	not found	not found	n/a
response	antimicrobial peptide		Alloferon		not found	not found	not found	not found	n/a
response	antimicrobial peptide		Andropin		not found	not found	not found	not found	n/a
response	antimicrobial peptide		Apisimin		not found	not found	not found	not found	n/a

Class	Role in Insect Immunity	Gene Symbol	Gene Name	Other Gene Names	ACYPI identifier	NCBI gene ID	NCBI transcript ID	NCBI protein ID	*Myzus* ID (e-value)
response	antimicrobial peptide	Att	Attacin		not found	not found	not found	not found	n/a
response	antimicrobial peptide	Cec	Cecropin		not found	not found	not found	not found	n/a
response	antimicrobial peptide		Coleoptericin		not found	not found	not found	not found	n/a
response	antimicrobial peptide	Def	Defensin		not found	not found	not found	not found	n/a
response	antimicrobial peptide		Diptericin		not found	not found	not found	not found	n/a
response	antimicrobial peptide		Drosocin		not found	not found	not found	not found	n/a
response	antimicrobial peptide		Drosomycin		not found	not found	not found	not found	n/a
response	antimicrobial peptide		Formicin		not found	not found	not found	not found	n/a
response	antimicrobial peptide		Gambicin		not found	not found	not found	not found	n/a
response	antimicrobial peptide		Gomesin		not found	not found	not found	not found	n/a
response	antimicrobial peptide		Heliocin		not found	not found	not found	not found	n/a
response	antimicrobial peptide		Holotricin		not found	not found	not found	not found	n/a
response	antimicrobial peptide		Lebocin		not found	not found	not found	not found	n/a
response	antimicrobial peptide		Megourin		not found, though see text	not found	not found	not found	n/a
response	antimicrobial peptide		Metchnikowin		not found	not found	not found	not found	n/a
response	antimicrobial peptide		Moricin		not found	not found	not found	not found	n/a
response	antimicrobial peptide		Penaeidin		not found, though see text	not found	not found	not found	n/a
response	antimicrobial peptide		Polyphemusin		not found	not found	not found	not found	n/a
response	antimicrobial peptide		Spingerin		not found	not found	not found	not found	n/a
response	antimicrobial peptide		Tachyplesin		not found	not found	not found	not found	n/a
response	antimicrobial peptide		Virescein		not found	not found	not found	not found	n/a
response	antimicrobial peptide	Thm1	Thaumatin1	Tha, Thn	ACYPI005841	100164856	XM_001942683.1	XP_001942718.1	81361 (1e-8)
response	antimicrobial peptide	Thm2	Thaumatin2	Tha, Thn	ACYPI001394	100160062	XM_001942537.1	XP_001942572.1	81361 (2e-8)
response	antimicrobial peptide	Thm3	Thaumatin3	Tha, Thn	ACYPI009605	100168942	XM_001942744.1	XP_001942779.1	81361 (2e-9)
response	antimicrobial peptide	Thm4	Thaumatin4	Tha, Thn	ACYPI003287	100162111	XM_001942495.1	XP_001942530.1	81361 (2e-9)
response	antimicrobial peptide	Thm5	Thaumatin5	Tha, Thn	ACYPI007568	100166717	XM_001951871.1	XP_001951906.1	9364 (5e-22)

Class	Role in Insect Immunity	Gene Symbol	Gene Name	Other Gene Names	ACYPI identifier	NCBI gene ID	NCBI transcript ID	NCBI protein ID	*Myzus* ID (e-value)
response	antimicrobial peptide	Thm6	Thaumatin6	Tha, Thn	ACYPI005301	100164271	XM_001942753.1	XP_001942788.1	81361 (3e-15)
response	microbial degradation	Lys1	Lysozyme, i-type		ACYPI002175	100160909	XM_001949053.1	XP_001949088.1	3094 (8e-63)
response	microbial degradation	Lys2	Lysozyme, i-type		ACYPI009125	100168424	XM_001949177.1	XP_001949212.1	584 (3e-80)
response	microbial degradation	Lys3	Lysozyme, i-type		ACYPI008509	100167742	XM_001949283.1	XP_001949318.1	74639 (7e-27)
response	fungal degradation	Cht1	Chitinase-like protein 1		ACYPI001365	100160032	XM_001943565.1	XP_001943600.1	3640 (0)
response	fungal degradation	Cht2	Chitinase-like protein 2	Drosophila chitinase 3	ACYPI010095	100169480	XM_001943003.1	XP_001943038.1	5202 (1e-52)
response	fungal degradation	Cht3	Chitinase-like protein 3		ACYPI001396	100160065	XM_001942561.1	XP_001942596.1	5202 (6e-73)
response	fungal degradation	Cht4	Chitinase-like protein 4		ACYPI006403	100165452	XM_001950345.1	XP_001950380.1	5202 (2e-168)
response	fungal degradation	Cht5	Chitinase-like protein 5		ACYPI009964	100169337	XM_001947381.1	XP_001947416.1	5202 (2e-52)
response	fungal degradation	Cht6	Chitinase-like protein 6		ACYPI009878	100169240	XM_001952683.1	XP_001952718.1	5202 (3e-50)
response	fungal degradation	Cht7	Chitinase-like protein 7		ACYPI005756	100164767	XM_001947852.1	XP_001947887.1	1327 (4e-162)
response	general stress response	TotA	Turandot A		not found	not found	not found	not found	n/a
response	general stress response	TotB	Turandot B		not found	not found	not found	not found	n/a
response	general stress response	TotC	Turandot C		not found	not found	not found	not found	n/a
response	general stress response	TotF	Turandot F		not found	not found	not found	not found	n/a
response	general stress response	TotM	Turandot M		not found	not found	not found	not found	n/a
response	general stress response	TotZ	Turandot Z		not found	not found	not found	not found	n/a
response	mark pathogens for phagocytosis	TepI	Thiolester containing protein I		not found	not found	not found	not found	n/a
response	mark pathogens for phagocytosis	TepII	Thiolester containing protein II		not found	not found	not found	not found	n/a
response	mark pathogens for phagocytosis	TepIII-1	Thiolester containing protein III – 1, partial		ACYPI005292	100164261	XM_001944313.1	XP_001944348.1	no hit
response	mark pathogens for phagocytosis	TepIII-2	Thiolester containing protein III – 2		ACYPI000145	100158705	XM_001945685.1	XP_001945720.1	no hit
response	mark pathogens for phagocytosis	TepIV	Thiolester containing protein IV		not found	not found	not found	not found	n/a
response	prophenoloxidase response	ProPO1	Prophenoloxidase 1	Diphenol oxidase A3	ACYPI001367	100160034	XM_001949272.1	XP_001949307.1	5431 (5e-147)
response	prophenoloxidase response	ProPO2	Prophenoloxidase 2	Diphenol oxidase A3	ACYPI004484	100163393	XM_001951102.1	XP_001951137.1	5721 (e3-133)
response	production of nitric oxide, a toxic gas	Nos	Nitric oxide synthase		ACYPI001689	100160390	XM_001946174.1	XP_001946209.1	462 (1e-35)

Class	Role in Insect Immunity	Gene Symbol	Gene Name	Other Gene Names	ACYPI identifier	NCBI gene ID	NCBI transcript ID	NCBI protein ID	*Myzus* ID (e-value)
response	general stress response	Hsc5	Heat shock cognate 5		ACYPI004693	100163620	XM_001950464.1	XP_001950499.1	
response	general stress response	Hsc70	Heat shock cognate 70		ACYPI007166	100166283	XM_001948031.1	XP_001948066.1	1624 (0)
response	general stress response	Hsc70-1	Heat shock cognate 70 - 1		ACYPI000474	100159065	XM_001951198.1	XP_001951233.1	182 (0)
response	general stress response	Hsc70-2	Heat shock cognate 70 - 2		ACYPI004809	100163748	XM_001951351.1	XP_001951386.1	182 (0)
response	general stress response	Hsc70Cb	Heat shock cognate 70 - Cb		ACYPI004544	100163455	XM_001951757.1	XP_001951792.1	2297 (0)
response	general stress response	Hsp14	Heat shock protein 14		ACYPI002719	100161502	XM_001945733.1	XP_001945768.1	3375 (0)
response	general stress response	Hsp21.4	Heat shock protein 21.4		ACYPI003907	100162777	XM_001949367.1	XP_001949402.1	6174 (2e-108)
response	general stress response	Hsp60	Heat shock protein 60		ACYPI009253	100168563	XM_001951338.1	XP_001951373.1	317 (6e-155)
response	general stress response	Hsp70Aa	Heat shock protein 70Aa		ACYPI009117	100168413	XM_001951880.1	XP_001951915.1	182 (0)
response	general stress response	Hsp70Ab	Heat shock protein 70Ab		ACYPI007961	100167145	XM_001949626.1	XP_001949661.1	182 (0)
response	general stress response	Hsp70Ba	Heat shock protein 70Ba		ACYPI008763	100168026	XM_001949802.1	XP_001949837.1	182 (0)
response	general stress response	Hsp83	Heat shock protein 83		ACYPI009380	100168702	XM_001944726.1	XP_001944761.1	1268 (0)
response	general stress response	Hsp83	Heat shock protein 83		ACYPI002010	100160736	XM_001943137.1	XP_001943172.1	1268 (0)
response	general stress response	Hsp90	Heat shock protein 90		ACYPI002398	100161155	XM_001951175.1	XP_001951210.1	5927 (1e-149)
response	general stress response	Hsp90	Heat shock protein 90		ACYPI009915	100169283	XM_001948902.1	XP_001948937.1	3803 (0)
response	detoxification	Gst	Glutathione S-transferase-like, microsomal		ACYPI004835	100163775	XM_001951200.1	XP_001951235.1	3849 (8e-76)
response	detoxification	Gst	Glutathione S-transferase-like, microsomal		ACYPI006691	100165764	XM_001946296.1	XP_001946331.1	3849 (5e-37)
response	detoxification	Gst	Glutathione S-transferase-like, sigma class		ACYPI000794	100159421	XM_001952064.1	XP_001952099.1	3513 (3e-99)
response	detoxification	Gst	Glutathione S-transferase-like, sigma class		ACYPI002127	100160859	XM_001952005.1	XP_001952040.1	3896 (1e-105)
response	detoxification	Gst	Glutathione S-transferase-like, sigma class		ACYPI002679	100161459	XM_001952021.1	XP_001952056.1	3896 (1e-100)
response	detoxification	Gst	Glutathione S-transferase-like, sigma class		ACYPI009326	100168645	XM_001952392.1	XP_001952427.1	1197 (6e-82)
response	detoxification	Gst	Glutathione S-transferase-like, sigma class		ACYPI009519	100168850	XM_001946569.1	XP_001946604.1	1197 (2e-102)
response	detoxification	Gst	Glutathione S-transferase-like, sigma class		ACYPI009520	100168850	XM_001946504.1	XP_001946539.1	1197 (2e-102)
response	detoxification	GstD	Glutathione S-transferase, delta class		ACYPI006899	100165990	XM_001951401.1	XP_001951436.1	2376 (4e-56)
response	detoxification	GstD	Glutathione S-transferase, delta class		ACYPI009586	100168923	XM_001952561.1	XP_001952596.1	2376 (5e-38)

Class	Role in Insect Immunity	Gene Symbol	Gene Name	Other Gene Names	ACYPI identifier	NCBI gene ID	NCBI transcript ID	NCBI protein ID	*Myzus* ID (e-value)
response	detoxification	GstD10	Glutathione S-transferase, delta class		ACYPI008042	100167231	XM_001948159.1	XP_001948194.1	2376 (7e-73)
response	detoxification	GstD4	Glutathione S-transferase, delta class		ACYPI001068	100159718	XM_001942679.1	XP_001942714.1	2376 (1e-84)
response	detoxification	GstD6	Glutathione S-transferase, delta class		ACYPI006598	100165666	XM_001952338.1	XP_001952373.1	2376 (4e-53)
response	detoxification	GstD6	Glutathione S-transferase, delta class, partial		ACYPI008550	100167788	XM_001952381.1	XP_001952416.1	2376 (3e-52)
response	detoxification	GstD8	Glutathione S-transferase, delta class		ACYPI008657	100167906	XM_001942576.1	XP_001942611.1	2376 (9e-112)
response	detoxification	GstD9	Glutathione S-transferase, delta class		ACYPI005620	100164626	XM_001950500.1	XP_001950535.1	569 (1e-123)
response	detoxification	Gst	Glutathione S-transferase, theta class		ACYPI007233	100166353	XM_001949321.1	XP_001949356.1	6981 (2e-79)
response	detoxification	Gst	Glutathione S-transferase, theta class		ACYPI009122	100168419	XM_001949359.1	XP_001949394.1	9615 (3e-73)
response	alarm pheromone production	IPPS	Isoprenyl diphosphate synthase		ACYPI000050	100144905	NM_001126161.3	NP_001119633.3	912 (0)
response	alarm pheromone production	FPPS	similar to Farnesyl diphosphate synthase 2		ACYPI007080	100166187	XM_001950388.1	XP_001950423.1	912 (0)

Table S2. Samples for qPCR expression study.

Sample Name	Sample Handling Notes
**Bacterial Stabbing Experiment**	
No stab control	Frozen 8hrs after exposure of treatment aphids
Sterile stab	Frozen 8hrs after exposure
E.coli stab	Frozen 8hrs after exposure
Gram- pathogen stab	Frozen 8hrs after exposure
Gram+ pathogen stab	Frozen 8hrs after exposure
**Bacterial Feeding Experiment**	
Feed control	Frozen 12hrs after exposure to diet
E. coli feed	Frozen 12hrs after exposure to diet
Gram- pathogen feed	Frozen 12hrs after exposure to diet
**Fungal Shower Experiment**	
Fungus control	Frozen 24hrs after exposure
Fungus infected	Frozen 24hrs after exposure
**Viral Stabbing Experiment**	
Virus control (no stab)	Frozen 16hrs after exposure
Virus infected	Frozen 16hrs after exposure
**Stress Experiment**	
No stress Control	Frozen at beginning of stress experiment
Alarm pheromone	Frozen minutes after exposure to EBF
Post alarm pheromone	Frozen 12hrs after exposure to EBF
Heat stress	Frozen immediately after 36°C heat shock
Post heat stress	Frozen 8hrs after being returned to 20°C
Starvation	Frozen 12hrs after being removed from plant

Table S3. Primers for qPCR expression study.

Gene symbol	Gene name	Putative function	ACYPI ID	Primer Pair (5' to 3')
GNBP2	gram-negative binding protein 2	recognition	ACYPI006143	gnbp2_1f: AATTTCCGTGATGGGTGTTTAAGT<br>gnbp2_1r: TTTGTTTTCATTCCATGTTGATGAC
Gale1	galectin 1	recognition and response	ACYPI001371	gale_1f: GCTCCAATACTCAATCCGACTCTT<br>gale_1r: CATCGTCCTTGTTTCAAACC
Tl	toll	toll signaling pathway	ACYPI000177	tl1_1f: GAGCTCACCGTTTAAACTTTGTCA<br>tl1_1r: CATCAACTGAACGAGCAATTTGA
Cact	cactus	toll signaling pathway	ACYPI006820	cact_1f: GATGGCCAAAGTGCTCTTCATT<br>cact_1r: GAGCTTTCATTAGGTGTTTCACAATTT
Dl	dorsal 1b	toll signaling pathway	ACYPI003588	dl_1f: CAAGAGAATAGAAAACCACATCGTCTA<br>dl_1r: AAACATCAGTGTTATGCGGCTAAG
DlB	dorsal 1b	toll signaling pathway	ACYPI005133	dlb_1f: CTCTCAGAGTACGAGAAGAAATAAGAGTAGAT<br>dlb_1r: AAACATCAGTGTTATGCGGCTAAG
Myd88	myeloid differentiation primary response gene	toll signaling pathway	ACYPI001638	myd88_1f: TGCATGTTAAATGCCACGAAA<br>myd88_1r: TCCTCTCCAATCCCTGGGTAA
IRD5	immune response deficient 5	imd/jnk pathway	ACYPI004933	IRD5_1f: TGCTTATCTTGCACCGGAAGT<br>IRD5_1r: ACTATGACTCCAACACTCCACATATCTAA
Bsk	basket (JNK)	jnk pathway	ACYPI004372	Basket_1f: TTTGATCGATTATTCCCTGATGTACT<br>Basket_1r: GTGCTTGGCTTGCTTTTAGTTTATT
JRA1	Jun-related antigen	imd/jnk pathway	ACYPI002386	Jra_1f: AAATCAAACTCGAAAGGAAAAGACA<br>Jra_1r: TTCGGCGGCATTTGGA
IAP2	inhibitor of apoptosis 2	imd/jnk pathway	ACYPI000445	Iap2_10f: TCGATGAACACAAACGTCACAA<br>Iap2_10r: GTTCACCAGTTTCCTTATGATTTTCAA
Stat92E-2	Signal-transducer and activator of transcription 2	Jak/Stat pathway	ACYPI005642	Stat2_37f: TCATTAGTTCAGTGGAAACGTCAAC<br>Stat2_37r: AATCACACAATTTCTCACACCAAGTT
Lys1	lysozyme, i-type	response to bacteria	ACYPI002175	lysoz1_1f: CGCACAGGACTGCAACCA<br>lysoz1_1r: GGATGGCCGCGTAATCAG
Lys2	lysozyme, i-type	response to bacteria	ACYPI009125	Lys2_10f: GCGTCAGAGACCCGTATTGC<br>Lys2_10r: GCAATCCTTTGCGTATCTCTGTATG
Lys3	lysozyme, i-type	response to bacteria	ACYPI008509	lysoz3_1f: CCGGTCAGTAGCAGAGGAAAGT<br>lysoz3_1r: ATGAGCTCTCGCGTAGTTTGG
Thm2	thaumatin 2	response (antimicrobial peptide)	ACYPI001394	Tha2_r1f: CAACAGTAAAGGAAAATGCGAAAC<br>Tha2_r1r: TGGCACGCCCATGATACC
Thm3	thaumatin 3	response (antimicrobial peptide)	ACYPI009605	Tha3_1f: GGGCAGGCAGGATTTGG<br>Tha3_1r: TTGGATCTTGTTCCCGCAAT
Thm4	thaumatin 4	response (antimicrobial peptide)	ACYPI003287	Tha4_1f: GGCGGGCAGGATTTGG<br>Tha4_1r: GTGGATCTTGTTCCCGCAAT
Thm6	thaumatin 6	response (antimicrobial peptide)	ACYPI005301	Tha6_1f: AAAATGCAGCGCTCAAGGA<br>Tha6_1r: CACTTGATCTTGTTCCCGCAAT
HSP60	heat shock protein 60	response to stress	ACYPI009253	hsp60_1f: GATGCAATGAACGACGAATATGTTA<br>hsp60_1r: CTGACAACTTTGGTTGGATCGA
HSC70	heat shock cognate 70	response to stress	ACYPI007166	HP3f: TTGGGTGGAGAAGATTTTGA<br>HP3r: ATGACTGGCAGAAAGACCAC
HSP83	heat shock protein 83	response to stress	ACYPI002010	HP6f: CCGTACTGATCCTGGTGAAC<br>HP6r: GCCAATGAATTGAGAGTGCT

Table S4. Expression of recognition and signaling genes. Values indicate the relative expression of a gene in a sample relative to expression in the appropriate control sample, +/- one standard deviation. For the bacterial stabbing experiment, we compared a sterile stab sample to a no stab sample only for genes that showed greater than 2-fold upregulation in one of the bacteria-stabbed samples. Relative expression values should be interpreted with caution as they are based on only a single experimental replicate pooling five aphids.

	GNBP2	GALE1	TOLL	CACTUS	DOR1	DOR1B	MYD88	IRD5	JNK	JRA1	IAP2	JAK	STAT92E2
No stab control	1.00+/-1.05	1.00+/-0.08	1.00+/-0.03	1.00+/-0.06	1.00+/-0.16	1.00+/-0.05	1.00+/-0.12	1.00+/-0.07	1.00+/-0.06	1.00+/-0.02	1.00+/-0.04	1.00+/-0.12	1.00+/-0.11
Sterile stab			3.01+/-0.07	0.66+/-0.02							0.48+/-0.03		
E.coli stab	0.49+/-0.05	0.66+/-0.02	5.53+/-0.48	2.18+/-0.13	1.66+/-0.07	0.72+/-0.03	0.82+/-0.06	0.45+/-0.04	0.87+/-0.05	1.19+/-0/03	1.64+/-0.08	0.84+/-0.78	0.46+/-0.04
Gram-pathogen stab	0.59+/-0.06	0.78+/-0.12	2.52+/-0.21	1.78+/-0.33	0.84+/-0.06	0.65+/-0.02	1.26+/-0.08	0.43+/-0.06	0.76+/-0.02	0.85+/-0.06	2.00+/-0.06	0.77+/-0.06	0.59+/-0.04
Gram+pathogen stab	0.82+/-0.04	0.69+/-0.02	2.28+/-0.22	1.42+/-0.06	0.89+/-0.05	0.51+/-0.05	0.99+/-0.05	0.51+/-0.05	0.69+/-0.04	0.91+/-0.05	2.43+/-0.16	0.73+/-0.04	0.42+/-0.02
Feed control	1.00+/-0.05	1.00+/-0.07	1.00+/-0.10	1.00+/-0.07	1.00+/-0.05	1.00+/-0.08	1.00+/-0.07	1.00+/-0.03	1.00+/-0.10	1.00+/-0.07	1.00+/-0.06	1.00+/-0.08	1.00+/-0.08
E. coli feed	1.01+/-0.05	0.77+/-0.05	1.45+/-0.19	2.06+/-0.12	0.88+/-0.07	0.80+/-0.05	0.91+/-0.03	1.00+/-0.03	0.72+/-0.05	1.04+/-0.07	1.15+/-0.06	0.77+/-0.02	0.74+/-0.10
Gram-pathogen feed	0.82+/-0.06	0.63+/-0.05	1.41+/-0.15	1.35+/-0.05	0.84+/-0.08	0.70+/-0.03	0.63+/-0.03	1.03+/-0.04	0.70+/-0.05	0.95+/-0.03	1.46+/-0.10	0.64+/-0.02	0.69+/-0.07
Fungus control	1.00+/-0.07	1.00+/-0.03	1.00+/-0.08	1.00+/-0.02	1.00+/-0.07	1.00+/-0.04	1.00+/-0.09	1.00+/-0.09	1.00+/-0.06	1.00+/-0.08	1.00+/-0.04	1.00+/-0.07	1.00+/-0.04
Fungus infected	0.91+/-0.03	1.20+/-0.18	1.16+/-0.10	0.87+/-0.11	1.06+/-0.02	1.00+/-0.07	1.00+/-0.05	0.62+/-0.07	1.30+/-0.10	1.00+/-0.03	0.88+/-0.03	1.32+/-0.07	1.32+/-0.09
Virus control	1.00+/-0.02	1.00+/-0.04	1.00+/-0.03	1.00+/-0.03	1.00+/-0.08	1.00+/-0.06	1.00+/-0.02	1.00+/-0.04	1.00+/-0.06	1.00+/-0.07	1.00+/-0.10	1.00+/-0.03	1.00+/-0.12
Virus infected	0.88+/-0.03	1.01+/-0.04	1.23+/-0.14	0.83+/-0.02	1.59+/-0.05	0.97+/-0.04	0.87+/-0.05	0.92+/-0.06	0.93+/-0.03	1.16+/-0.06	0.98+/-0.07	1.40+/-0.11	2.42+/-0.27
No stress control	1.00+/-0.10	1.00+/-0.14	1.00+/-0.03	1.00+/-0.02	1.00+/-0.07	1.00+/-0.07	1.00+/-0.04	1.00+/-0.06	1.00+/-0.04	1.00+/-0.04	1.00+/-0.08	1.00+/-0.06	1.00+/-0.06
Alarm pheromone	0.79+/-0.05	0.80+/-0.07	0.94+/-0.03	2.41+/-0.24	0.96+/-0.05	0.87+/-0.04	0.74+/-0.02	0.95+/-0.13	0.97+/-0.06	0.96+/-0.03	1.37+/-0.09	1.15+/-0.07	0.98+/-0.05
Post alarm pheromone	0.54+/-0.03	0.94+/-0.12	0.34+/-0.05	2.46+/-0.10	0.78+/-0.15	0.98+/-0.07	0.96+/-0.07	1.03+/-0.14	0.99+/-0.06	0.64+/-0.13	2.11+/-0.14	1.08+/-0.08	1.00+/-0.07
Heat stress	1.35+/-0.07	1.14+/-0.05	0.74+/-0.08	1.48+/-0.06	2.59+/-0.17	1.71+/-0.06	1.01+/-0.03	0.94+/-0.07	1.14+/-0.07	1.17+/-0.16	0.68+/-0.02	1.27+/-0.22	1.97+/-0.12
Post heat stress	0.76+/-0.04	0.69+/-0.09	0.48+/-0.02	1.68+/-0.10	0.75+/-0.11	0.63+/-0.08	0.58+/-0.03	0.67+/-0.04	0.77+/-0.01	0.69+/-0.02	2.11+/-0.12	0.76+/-0.02	0.58+/-0.07
Starvation	2.14+/-0.42	1.16+/-0.03	0.64+/-0.05	3.29+/-0.27	1.79+/-0.12	1.36+/-0.06	0.76+/-0.06	0.86+/-0.11	1.20+/-0.06	0.44+/-0.08	1.13+/-0.14	1.37+/-0.07	1.03+/-0.12

Table S5. Expression of response genes. Values indicate the relative expression of a gene in a sample relative to expression in the appropriate control sample, +/- S.D. For the bacterial stabbing experiment, we compared a sterile stab sample to a no stab sample only for genes that showed greater than 2-fold upregulation in one of the bacteria-stabbed samples. Relative expression values should be interpreted with caution as they are based on only a single experimental replicate pooling five aphids.

	LYS1	LYS2	LYS3	THM2	THM3	THM4	THM6	HSP60	HSC70	HSP83
No stab control	1.00+/-0.05	1.00+/-0.07	1.00+/-0.03	1.00+/-0.04	1.00+/-0.09	1.00+/-0.06	1.00+/-0.06	1.00+/-0.05	1.00+/-0.05	1.00+/-0.05
Sterile stab				0.74+/-0.07						
E.coli stab	1.00+/-0.03	1.35+/-0.08	1.76+/-0.15	2.37+/-0.07	0.34+/-0.05	0.29+/-0.07	0.39+/-0.03	0.81+/-0.04	0.58+/-0.04	0.80+/-0.06
Gram-pathogen stab	1.12+/-0.07	1.41+/-0.05	1.57+/-0.07	1.80+/-0.03	1.06+/-0.04	0.86+/-0.08	1.60+/-0.06	0.96+/-0.05	0.69+/-0.05	1.26+/-0.07
Gram+pathogen stab	1.12+/-0.04	1.59+/-0.17	1.62+/-0.06	0.56+/-0.05	0.58+/-0.04	0.51+/-0.04	1.01+/-0.07	0.84+/-0.04	0.66+/-0.02	0.97+/-0.04
Feed control	1.00+/-0.02	1.00+/-0.12	1.00+/-0.04	1.00+/-0.04	1.00+/-0.05	1.00+/-0.05	1.00+/-0.06	1.00+/-0.09	1.00+/-0.04	1.00+/-0.07
E. coli feed	2.16+/-0.13	1.17+/-0.05	1.18+/-0.12	0.77+/-0.06	0.67+/-0.05	0.69+/-0.04	0.59+/-0.02	1.17+/-0.14	0.80+/-0.05	0.77+/-0.03
Gram-pathogen feed	2.66+/-0.22	0.91+/-0.05	1.09+/-0.02	0.30+/-0.08	0.39+/-0.02	0.41+/-0.02	0.67+/-0.03	0.88+/-0.05	0.76+/-0.03	0.64+/-0.03
Fungus control	1.00+/-0.01	1.00+/-0.10	1.00+/-0.05	1.00+/-0.11	1.00+/-0.06	1.00+/-0.07	1.00+/-0.03	1.00+/-0.05	1.00+/-0.04	1.00+/-0.01
Fungus infected	0.87+/-0.15	0.78+/-0.03	0.85+/-0.05	1.10+/-0.30	1.07+/-0.06	1.30+/-0.06	1.43+/-0.06	0.92+/-0.19	0.97+/-0.09	2.31+/-0.06
Virus control	1.00+/-0.04	1.00+/-0.02	1.00+/-0.02	1.00+/-0.14	1.00+/-0.16	1.00+/-0.12	1.00+/-0.10	1.00+/-0.06	1.00+/-0.02	1.00+/-0.02
Virus infected	0.92+/-0.04	0.67+/-0.03	0.64+/-0.02	0.64+/-0.06	0.95+/-0.14	0.96+/-0.11	0.93+/-0.13	0.74+/-0.02	0.77+/-0.07	1.20+/-0.06
No stress control	1.00+/-0.02	1.00+/-0.05	1.00+/-0.02	1.00+/-0.06	1.00+/-0.11	1.00+/-0.04	1.00+/-0.05	1.00+/-0.08	1.00+/-0.07	1.00+/-0.05
Alarm pheromone	0.87+/-0.13	0.92+/-0.03	0.86+/-0.03	0.30+/-0.04	0.49+/-0.01	1.05+/-0.04	1.37+/-0.05	0.77+/-0.02	1.14+/-0.02	1.21+/-0.05
Post alarm pheromone	0.89+/-0.04	0.76+/-0.04	0.93+/-0.01	0.34+/-0.07	0.64+/-0.03	0.65+/-0.05	0.22+/-0.05	1.44+/-0.15	1.20+/-0.05	1.28+/-0.03
Heat stress	1.48+/-0.04	0.98+/-0.05	0.66+/-0.02	1.70+/-0.10	0.95+/-0.05	0.68+/-0.02	0.35+/-0.06	1.55+/-0.09	2.07+/-0.06	5.54+/-0.27
Post heat stress	0.78+/-0.13	0.61+/-0.02	0.67+/-0.04	0.31+/-0.07	0.85+/-0.04	0.74+/-0.06	0.64+/-0.05	0.92+/-0.02	1.01+/-0.07	0.26+/-0.01
Starvation	1.30+/-0.11	2.96+/-0.23	1.19+/-0.06	0.75+/-0.02	0.90+/-0.05	0.93+/-0.04	0.53+/-0.04	1.09+/-0.08	1.02+/-0.10	0.64+/-0.04

Table S6. Gut EST library statistics.

	TD2a library	TD2b library
Library ID	ID0AFF	ID0AAG
Tissue treatment	Control digestive tract	Gram – challenged digestive tract
	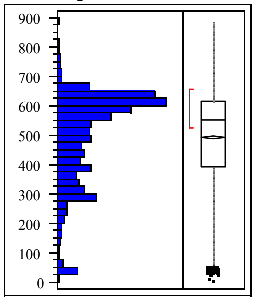	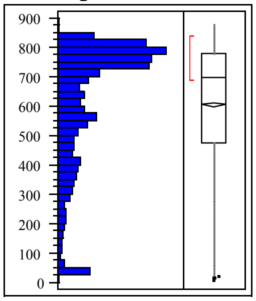
Number of clones sequenced	5283	4043
Mean clone length	490 ± 160	602 ± 219
Median clone length	548	694
N seq. < 100 bp	210	197
Clones with no blastX hit (E≥10) [a]	737	633
% without hit	14,0%	15,7%
Clones with blastX hit (E<10)	4546 (4065*)	3410 (2921*)
Clones with non-sign. hits (10^{-3} – 10)	1408	936
Clones with sign. hits (E ≤ 10^{-3})	3138	2474
% with sign. hit	59,4%	61,2%
Uniprot hits from blastX (total)	2192	806
Uniprot hits from blastX (significant)	1486	578
N hits with more than 10 clones	56	50
N hits with more than 1% expression	6	12
N hits with more than 2% expression	1	9
Hits with GO annotation (%) [b]	79 %	80 %
Hits without GO annotation (total Fatigo)	312 (1495)	117 (579)
N Contigs (Ap v5 clustering)	2128	875
redundancy index (% contigs vs clones)	40.3 %	21.6 %
Nb specific contigs (control *vs* challenged)	1724	471
specificity rate (% specific vs total)	81%	54%
Fatigo analysis, total genes analysed	1495	579
Go class (4th level) [c]		
biopolymer metabolism (*0.005* – 0.088)	**21.0 %**	**11.3 %**
transport (*0.029* – 0.45)	**19.1 %**	**27.1 %**
establishment of localization (*0.038* – 0.64)	**19.2 %**	**27.0 %**
cellular metabolism	**78.4 %**	**71.7 %**
cell organization and biogenesis	**7.4 %**	**3.8 %**
primary metabolism	**67.7 %**	**62.3 %**
macromolecule metabolism	**46.9 %**	**44.0 %**
biosynthesis	**24.3 %**	**26.4 %**
catabolism	**14.3 %**	**12.6 %**

a : blastX performed through the blast parsing script runblastncbi (courtesy Laurent Duret), on Uniprot DB (May 2005, release UniprotKB 4.0, pbil server pbil.univ-lyon1.fr)
b : FATIGO links to GO ontology (June 2005, UniprotKB release 4.0, www.fatigo.org)
c : beneath % of class are given the *unadjusted* and adjusted (step-down min p) p values for library comparison, as computed by Fatigo (Fisher exact test). For non-discrimnant classes (no p given, p≥10%), only main classes are listed (>10% representation)
* : corrected for hit redundancy in blast results (low complexity or modular proteins)

Table S7. List of selected ESTs from the subtracted library. Results include the highest homologous match for each EST against the *A. pisum* NCBI refseq set (blastX), the publicly available *A. Pisum* ESTs (blastN), and the SwissProt databases (blastX). ESTs have been selected according to either apparent differential gene expression or to their similarities to putative immune-related genes. Differential expression status of ESTs was analyzed using a dot-blot experiment as described in the materials and methods. The change in gene expression after *E. coli* infection relative to the untreated aphid is given as qualitative data (+: overexpression; -: underexpression; =: no differential gene expression). Descriptions for aphid matches are based on *A. pisum* EST cluster descriptions, available at the *Acyrthosiphon Pisum* EST Database [142], and/or on refseq descriptions.

EST			Aphid Hits				Swiss Prot Hits		
GenBank Acc. Num.	Length	Relative expression	*A. pisum* gene (evalue)	*A. pisum* EST (evalue)	*A. pisum* EST cluster	Description based on gene or EST	Swiss Prot ID (evalue)	Organism	Description
GD186025	237	-	no hit	EX619631 (e-104)	APG20589		no hit		
GD186052	629	-	ACYPI006141 (5e-31)	FF333609 (0.0)	APG03947	similar to eukaryotic translation initiation factor 5	Q9VXK6 (9e-26)	*Drosophila melanogaster*	eukaryotic translation initiation factor 5
GD186090	299	-	ACYPI004024 (3e-11)	FF338507 (e-106)	APG01676	transport, small GTPase mediated signal transduction	Q9VZ23 (2e-07)	*Drosophila melanogaster*	GTP-binding nuclear protein Ran
GD186102	425	-	no hit	DY224167	APD07979		no hit		
GD185918	445	+	no hit	FF317856	APG05151		no hit		
GD185990	539	+	ACYPI005949 (5e-99)	FF334362 (0.0)	APG08004	Rps3 ribosomal protein S3	P48153 (2e-81)	*Manduca sexta*	40S ribosomal protein S3
GD186053	320	+	ACYPI006749 (8e-34)	EX620733 (e-169)	APG08261	methionine biosynthesis	no hit		
GD186057	748	+	ACYPI006004 (2e-189)	FF324610 (0.0)	APG11294	similar to latent nuclear antigen	Q8IMP6 (7e-23)	*Drosophila melanogaster*	protein SPT2 homolog
GD186146	540	+	ACYPI003334 (6e-62)	EX607065 (0.0)	APG09767		Q6GMF8 (5e-46)	*Danio rerio*	rhomboid family member 1
GD186208	397	+	no hit	FF317879 (0.0)	APG01972	nuclear mRNA splicing	no hit		
GD186213	399	+	no hit	EX653226 (e-113)	APG17996		no hit		
GD186220	314	+	ACYPI000343 (8e-59)	CN762809 (e-155)	APD04091	similar to ubiquitin specific protease 7	Q9VYQ8 (5e-17)	*Drosophila melanogaster*	ubiquitin carboxyl-terminal hydrolase 7
GD186223	571	+	ACYPI007035 (4e-85)	FF317872 (0.0)	APG06079	similar to coiled-coil domain-containing protein 132	Q8CI71 (1e-19)	*Mus musculus*	coiled-coil domain-containing protein 132
GD186225	333	+	no hit	no hit			no hit		
GD186231	379	+	ACYPI003578 (2e-58)	FF314239 (0.0)	APG03794	similar to signal recognition particle 72 kDa protein	P33731 (7e-15)	*Canis lupus familiaris*	signal recognition particle 72 kDa protein
GD186337	674	+	no hit	FF333448 (e-115)	APG13156	amino acid transport	Q54S12 (0.12)	*Dictyostelium discoideum*	transmembrane protein 104 homolog
GD186362	397	+	no hit	DY223529 (e-103)	APD03776		no hit		
GD186383	375	+	no hit	FF305066 (0.0)	APG03552	protein amino acid phosphorylation	Q16513 (0.003)	*Homo sapiens*	serine/threonine-protein kinase N2
GD186386	361	+	no hit	FF330011	APG09318		no hit		

EST			Aphid Hits				Swiss Prot Hits		
GenBank Acc. Num.	Length	Relative expression	*A. pisum* gene (evalue)	*A. pisum* EST (evalue)	*A. pisum* EST cluster	Description based on gene or EST	Swiss Prot ID (evalue)	Organism	Description
				(e-115)					
GD185976	283	=	ACYPI002027 (2e-58)	CV838512 (e-154)	APD02867	thrombospondin-like	Q9C0I4 (1e-16)	*Homo sapiens*	thrombospondin type-1 domain-containing protein 7B
GD186047	347	=	ACYPI000111 (1e-68)	no hit		similar to adam	Q9VAC5 (2e-53)	*Drosophila melanogaster*	Adam 17-like protease
GD186063	1130	=	ACYPI000719 (3e-136)	FF336689 (0.0)	APG09006		P13284 (7e-13)	*Homo sapiens*	gamma-interferon-inducible lysosomal thiol reductase precursor
GD186100	284	=	ACYPI008118 (4e-102)	FF315088 (0.0)	APG16211	similar to tyrosine protein kinase	Q24592 (2e-08)	*Drosophila melanogaster*	tyrosine-protein kinase hopscotch (Jak)
GD186119	325	=	ACYPI005016 (2e-51)	FF320908 (e-176)	APG08844	serine protease inhibitor (serpin 1)	P48594 (2e-17)	*Homo sapiens*	serpin B4
GD186120	353	=	ACYPI005540 (6e-63)	CN759023 (0.0)	APD04504	MAPKKK cascade	Q5E9X2 (3e-45)	*Bos taurus*	dual specificity mitogen-activated protein kinase 6
GD186131	517	=	ACYPI005016 (5e-86)	FF336182 (0.0)	APG07692	serine-protease inhibitor (serpin 1)	P48594 (2e-17)	*Homo sapiens*	serpin B4
GD186142	328	=	no hit	EX639251 (e-110)	APG19100	receptor activity	Q5XIN3 (1e-07)	*Rattus norvegicus*	TRAF3-interacting protein 1
GD186162	330	=	ACYPI003236 (3e-59)	no hit		similar to sunday driver	Q9ESN9 (9e-34)	*Mus musculus*	JNK-interacting protein 3
GD186173	245	=	no hit	CN761251 (e-119)	APD04293	programs cell death	Q3UU35 (0.8)	*Mus musculus*	Ovostatin homolog
GD186257	345	=	ACYPI006651 (7e-32)	CN764436 (e-163)	APD05533	similar to membrane-associated LPS-inducible TNF alpha factor	Q8QGW7 (7e-10)	*Gallus gallus*	LPS-induced TNF-alpha factor homolog
GD186266	346	=	ACYPI002465 (3e-28)	CN756373 (e-173)	APD05909	similar to macrophage migration inhibitory factor	P91850 (3e-08)	*Brugia malayi*	Macrophage migration inhibitory factor homolog
GD186363	268	=	no hit	EX650498 (e-126)	APG05258	sugar binding	no hit		

Figure S1. Maximum likelihood phylogenies of selected immune gene families. (a) Gram-negative binding proteins; (b) Spätzles; (c) Tolls, note that there is no support for *A. pisum* tolls clading with *D. melanogaster* Tehao; (d) Lysozymes; (e) Teps; (f) Prophenoloxidases; and, (g) low molecular weight heat shock proteins (Hsp83, Hsp90 not included). * represents approximate likelihood ratio test (aLRT) support > 80. (Aga: *Anopheles gambiae*; Ame: *Apis Mellifera*; Api: *Acythrosiphon pisum*; Bom: *Bombyx mori*; Cel: *Caenorhabditis elegans*; Cin: *Ciona_intestinalis*; Dme = *Drosophila melanogaster*: Dpu = *Daphnia pulex*; Nvi: *Nasonia vitripennis*; Phu: *Pediculus humanus*; Tca: *Tribolium castaneum*.)

(a)

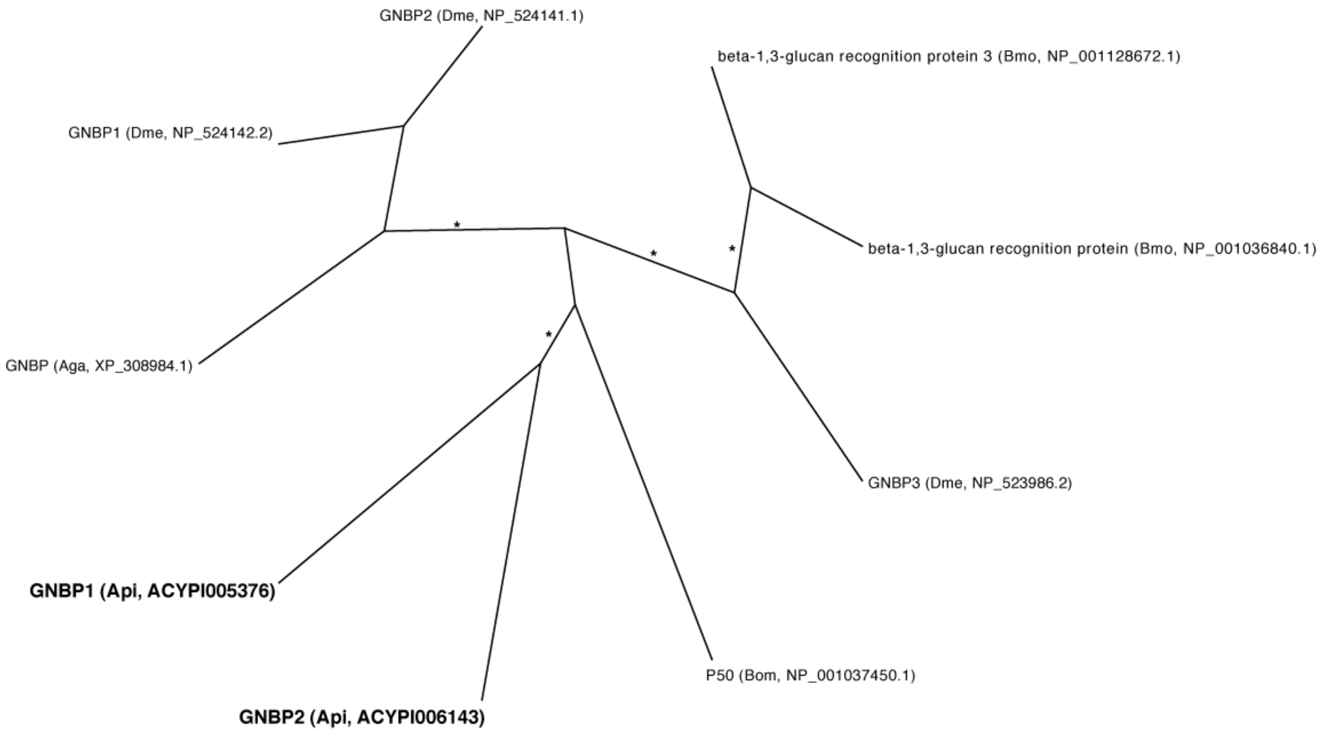

(b)

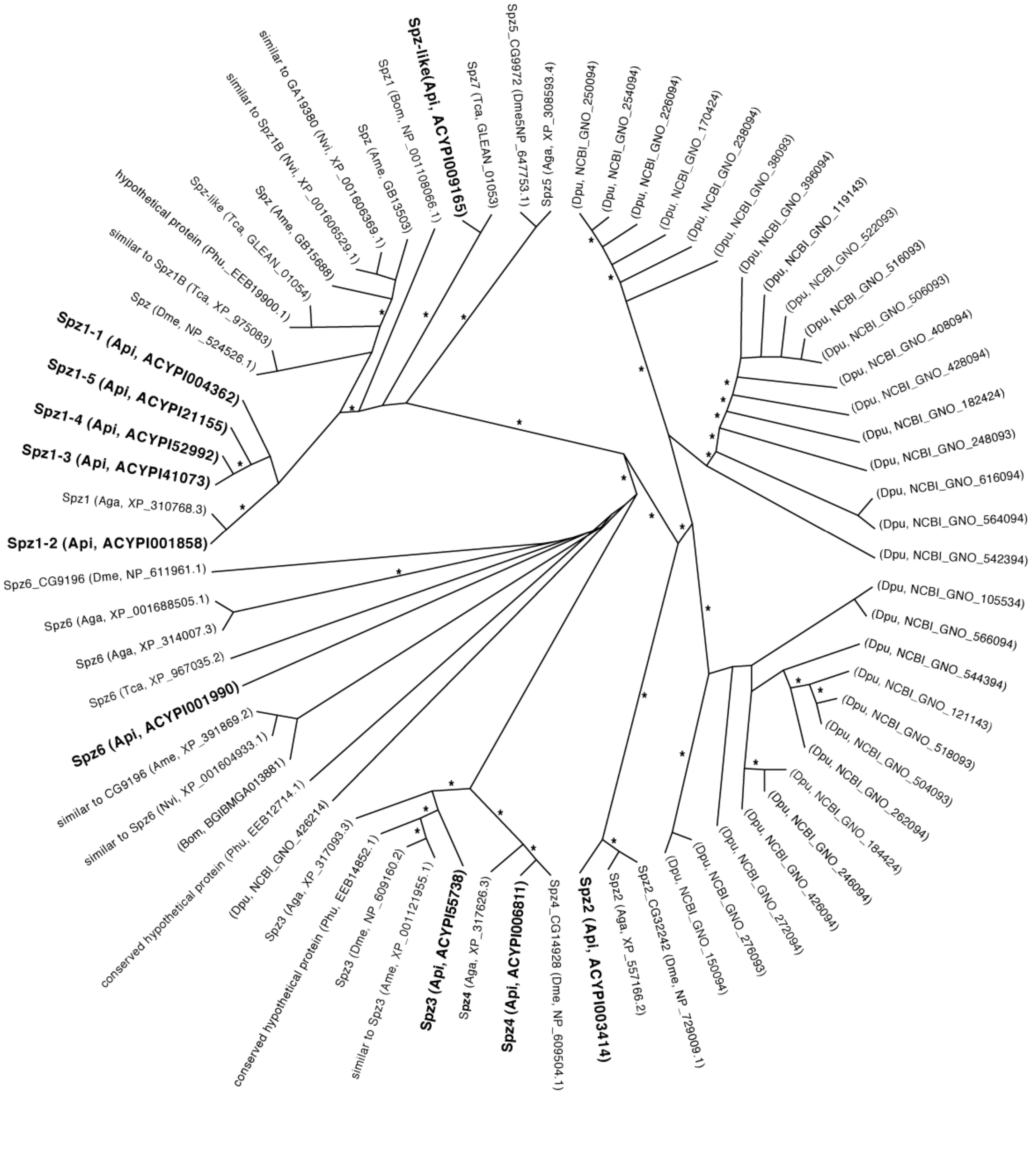
Spz5_CG9972 (Dme5NP_647753.1)
Spz5 (Aga, XP_308593.4)
Spz7 (Tca, GLEAN_01053)
Spz-like(Api, ACYPI009165)
Spz1 (Bom, NP_001108066.1)
similar to GA19380 (Nvi, XP_001606369.1)
Spz (Ame, GB13503)
similar to Spz1B (Nvi, XP_001606529.1)
Spz (Ame, GB15688)
Spz-like (Tca, GLEAN_01054)
hypothetical protein (Phu, EEB19900.1)
similar to Spz1B (Tca, XP_975083)
Spz (Dme, NP_524526.1)
Spz1-1 (Api, ACYPI004362)
Spz1-5 (Api, ACYPI21155)
Spz1-4 (Api, ACYPI52992)
Spz1-3 (Api, ACYPI41073)
Spz1 (Aga, XP_310768.3)
Spz1-2 (Api, ACYPI001858)
Spz6_CG9196 (Dme, NP_611961.1)
Spz6 (Aga, XP_001688505.1)
Spz6 (Aga, XP_314007.3)
Spz6 (Tca, XP_967035.2)
Spz6 (Api, ACYPI001990)
similar to CG9196 (Ame, XP_391869.2)
similar to Spz6 (Nvi, XP_001604933.1)
(Bom, BGIBMGA013881)
conserved hypothetical protein (Phu, EEB12714.1)
(Dpu, NCBI_GNO_426214)
Spz3 (Aga, XP_317093.3)
conserved hypothetical protein (Phu, EEB14852.1)
Spz3 (Dme, NP_609160.2)
similar to Spz3 (Ame, XP_001121955.1)
Spz3 (Api, ACYPI55738)
Spz4 (Aga, XP_317626.3)
Spz4 (Api, ACYPI006811)
Spz4_CG14928 (Dme, NP_609504.1)
Spz2 (Api, ACYPI003414)
Spz2 (Aga, XP_557166.2)
Spz2_CG32242 (Dme, NP_729009.1)
(Dpu, NCBI_GNO_150094)
(Dpu, NCBI_GNO_276093)
(Dpu, NCBI_GNO_272094)
(Dpu, NCBI_GNO_426094)
(Dpu, NCBI_GNO_246094)
(Dpu, NCBI_GNO_184424)
(Dpu, NCBI_GNO_262094)
(Dpu, NCBI_GNO_504093)
(Dpu, NCBI_GNO_518093)
(Dpu, NCBI_GNO_121143)
(Dpu, NCBI_GNO_544394)
(Dpu, NCBI_GNO_566094)
(Dpu, NCBI_GNO_105534)
(Dpu, NCBI_GNO_542394)
(Dpu, NCBI_GNO_564094)
(Dpu, NCBI_GNO_616094)
(Dpu, NCBI_GNO_248093)
(Dpu, NCBI_GNO_182424)
(Dpu, NCBI_GNO_428094)
(Dpu, NCBI_GNO_408094)
(Dpu, NCBI_GNO_506093)
(Dpu, NCBI_GNO_516093)
(Dpu, NCBI_GNO_522093)
(Dpu, NCBI_GNO_119143)
(Dpu, NCBI_GNO_396094)
(Dpu, NCBI_GNO_38093)
(Dpu, NCBI_GNO_238094)
(Dpu, NCBI_GNO_170424)
(Dpu, NCBI_GNO_226094)
(Dpu, NCBI_GNO_254094)
(Dpu, NCBI_GNO_250094)

(c)

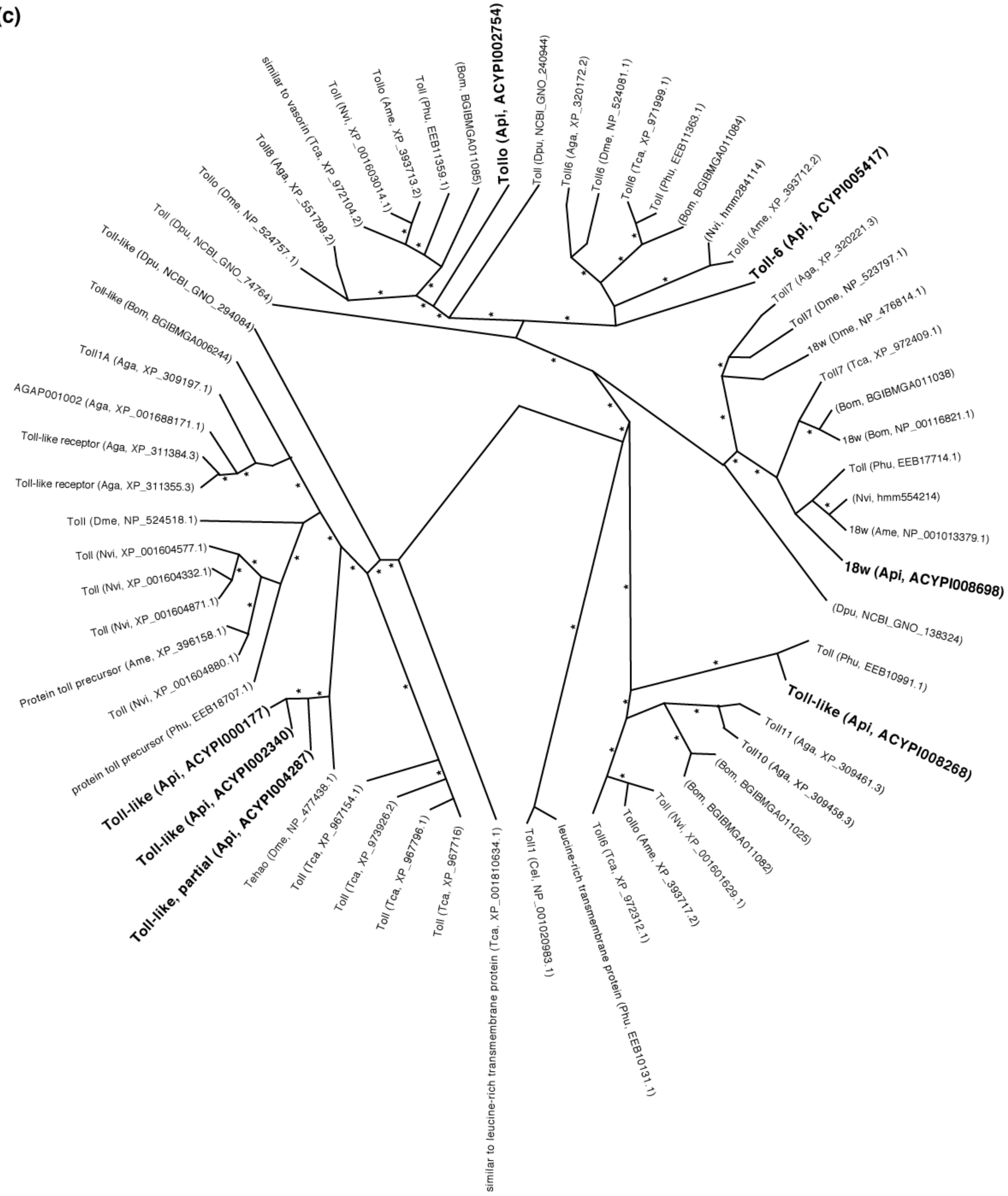

Tollo (Api, ACYPI002754)
Toll-6 (Api, ACYPI005417)
18w (Api, ACYPI008698)
Toll-like (Api, ACYPI008268)
Toll-like (Api, ACYPI000177)
Toll-like (Api, ACYPI002340)
Toll-like, partial (Api, ACYPI004287)
Toll (Dme, NP_524518.1)
Toll-like receptor (Aga, XP_311355.3)
Toll-like receptor (Aga, XP_311384.3)
AGAP001002 (Aga, XP_001688171.1)
Toll1A (Aga, XP_309197.1)
Toll-like (Bom, BGIBMGA006244)
Toll-like (Dpu, NCBI_GNO_294084)
Toll (Dpu, NCBI_GNO_74764)
Tollo (Dme, NP_524757.1)
Toll8 (Aga, XP_551799.2)
similar to vasorin (Tca, XP_972104.2)
Toll (Nvi, XP_001603014.1)
Tollo (Ame, XP_393713.2)
Toll (Phu, EEB11359.1)
(Bom, BGIBMGA011085)
Toll (Dpu, NCBI_GNO_240944)
Toll6 (Aga, XP_320172.2)
Toll6 (Dme, NP_524081.1)
Toll6 (Tca, XP_971999.1)
Toll (Phu, EEB11363.1)
(Bom, BGIBMGA011084)
(Nvi, hmm284114)
Toll6 (Ame, XP_393712.2)
Toll7 (Aga, XP_320221.3)
Toll7 (Dme, NP_523797.1)
18w (Dme, NP_476814.1)
Toll7 (Tca, XP_972409.1)
(Bom, BGIBMGA011038)
18w (Bom, NP_00116821.1)
Toll (Phu, EEB17714.1)
(Nvi, hmm554214)
18w (Ame, NP_001013379.1)
(Dpu, NCBI_GNO_138324)
Toll (Phu, EEB10991.1)
Toll11 (Aga, XP_309461.3)
Toll10 (Aga, XP_309458.3)
(Bom, BGIBMGA011025)
(Bom, BGIBMGA011082)
Toll (Nvi, XP_001601629.1)
Tollo (Ame, XP_393717.2)
Toll6 (Tca, XP_972312.1)
leucine-rich transmembrane protein (Phu, EEB10131.1)
Toll1 (Cel, NP_001020983.1)
similar to leucine-rich transmembrane protein (Tca, XP_001810634.1)
Toll (Tca, XP_967716)
Toll (Tca, XP_967796.1)
Toll (Tca, XP_973926.2)
Toll (Tca, XP_967154.1)
Tehao (Dme, NP_477438.1)
protein toll precursor (Phu, EEB18707.1)
Toll (Nvi, XP_001604880.1)
Protein toll precursor (Ame, XP_396158.1)
Toll (Nvi, XP_001604871.1)
Toll (Nvi, XP_001604332.1)
Toll (Nvi, XP_001604577.1)

(d)

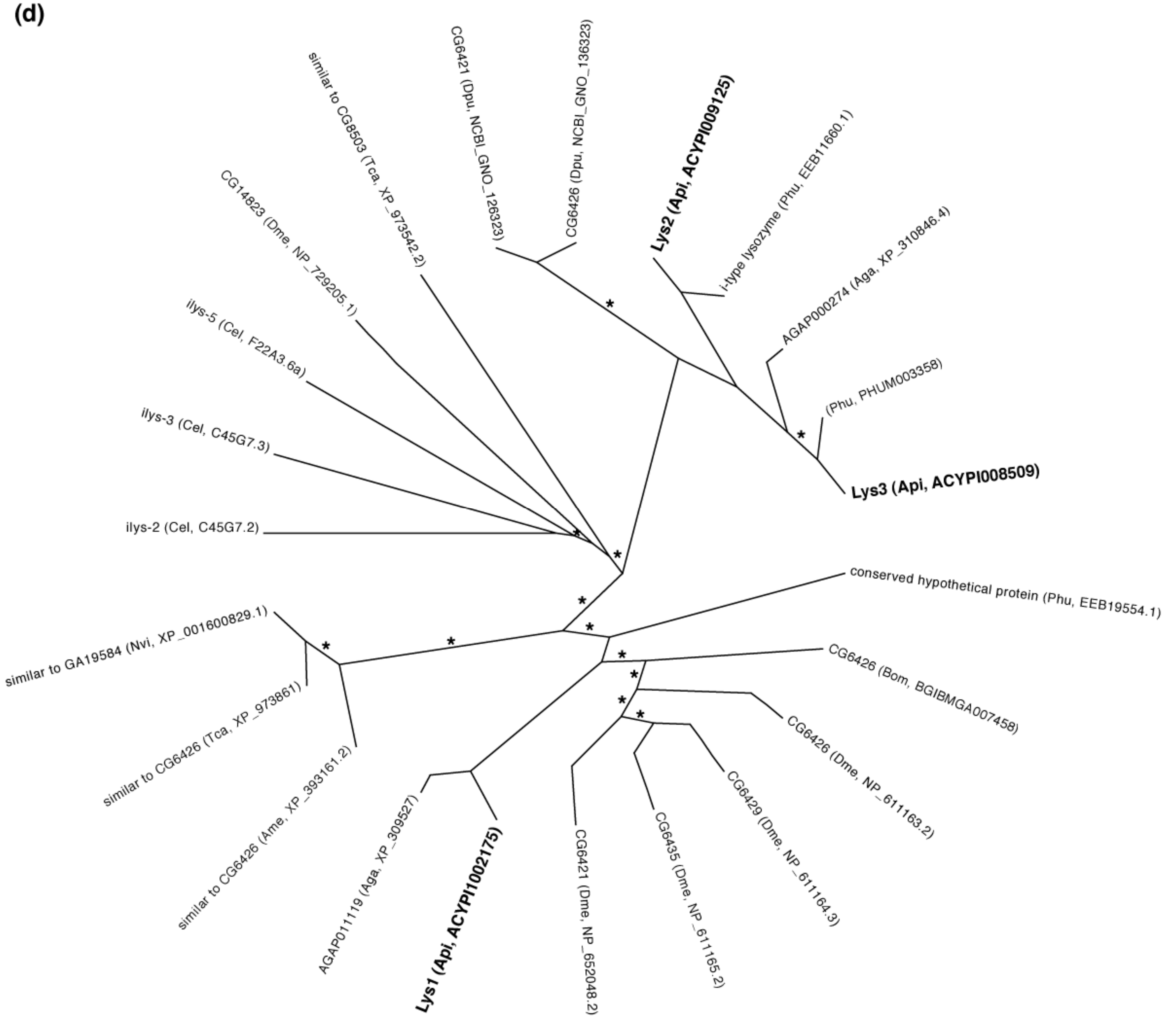

similar to CG8503 (Tca, XP_973542.2)
CG6421 (Dpu, NCBI_GNO_126323)
CG6426 (Dpu, NCBI_GNO_136323)
Lys2 (Api, ACYPI009125)
i-type lysozyme (Phu, EEB11660.1)
AGAP000274 (Aga, XP_310846.4)
(Phu, PHUM003358)
Lys3 (Api, ACYPI008509)
CG14823 (Dme, NP_729205.1)
ilys-5 (Cel, F22A3.6a)
ilys-3 (Cel, C45G7.3)
ilys-2 (Cel, C45G7.2)
conserved hypothetical protein (Phu, EEB19554.1)
similar to GA19584 (Nvi, XP_001600829.1)
CG6426 (Bom, BGIBMGA007458)
similar to CG6426 (Tca, XP_973861)
CG6426 (Dme, NP_611163.2)
similar to CG6426 (Ame, XP_393161.2)
AGAP011119 (Aga, XP_309527)
Lys1 (Api, ACYPI002175)
CG6421 (Dme, NP_652048.2)
CG6435 (Dme, NP_611165.2)
CG6429 (Dme, NP_611164.3)

(e)

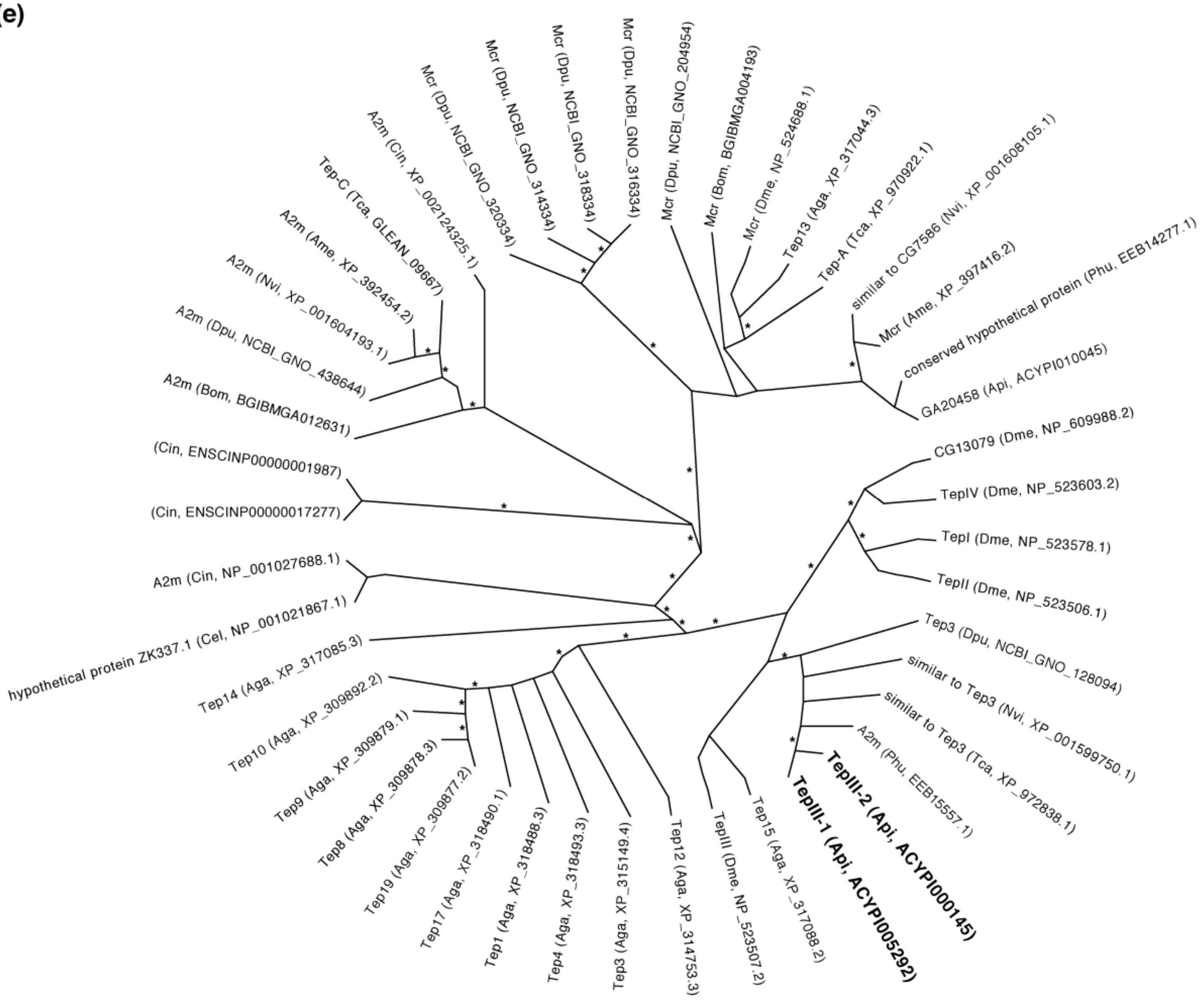
Mcr (Dpu, NCBI_GNO_320334)
Mcr (Dpu, NCBI_GNO_314334)
Mcr (Dpu, NCBI_GNO_318334)
Mcr (Dpu, NCBI_GNO_316334)
Mcr (Dpu, NCBI_GNO_204954)
Mcr (Bom, BGIBMGA004193)
Mcr (Dme, NP_524688.1)
Tep13 (Aga, XP_317044.3)
Tep-A (Tca, XP_970922.1)
similar to CG7586 (Nvi, XP_001608105.1)
Mcr (Ame, XP_397416.2)
conserved hypothetical protein (Phu, EEB14277.1)
GA20458 (Api, ACYPI010045)
CG13079 (Dme, NP_609988.2)
TepIV (Dme, NP_523603.2)
TepI (Dme, NP_523578.1)
TepII (Dme, NP_523506.1)
Tep3 (Dpu, NCBI_GNO_128094)
similar to Tep3 (Nvi, XP_001599750.1)
similar to Tep3 (Tca, XP_972838.1)
A2m (Phu, EEB15557.1)
TepIII-2 (Api, ACYPI000145)
TepIII-1 (Api, ACYPI005292)
Tep15 (Aga, XP_317088.2)
TepIII (Dme, NP_523507.2)
Tep12 (Aga, XP_314753.3)
Tep3 (Aga, XP_315149.4)
Tep4 (Aga, XP_318493.3)
Tep1 (Aga, XP_318488.3)
Tep17 (Aga, XP_318490.1)
Tep19 (Aga, XP_309877.2)
Tep8 (Aga, XP_309878.3)
Tep9 (Aga, XP_309879.1)
Tep10 (Aga, XP_309892.2)
Tep14 (Aga, XP_317085.3)
hypothetical protein ZK337.1 (Cel, NP_001021867.1)
A2m (Cin, NP_001027688.1)
(Cin, ENSCINP00000017277)
(Cin, ENSCINP00000001987)
A2m (Bom, BGIBMGA012631)
A2m (Dpu, NCBI_GNO_438644)
A2m (Nvi, XP_001604193.1)
A2m (Ame, XP_392454.2)
Tep-C (Tca, GLEAN_09667)
A2m (Cin, XP_002124325.1)

(f)

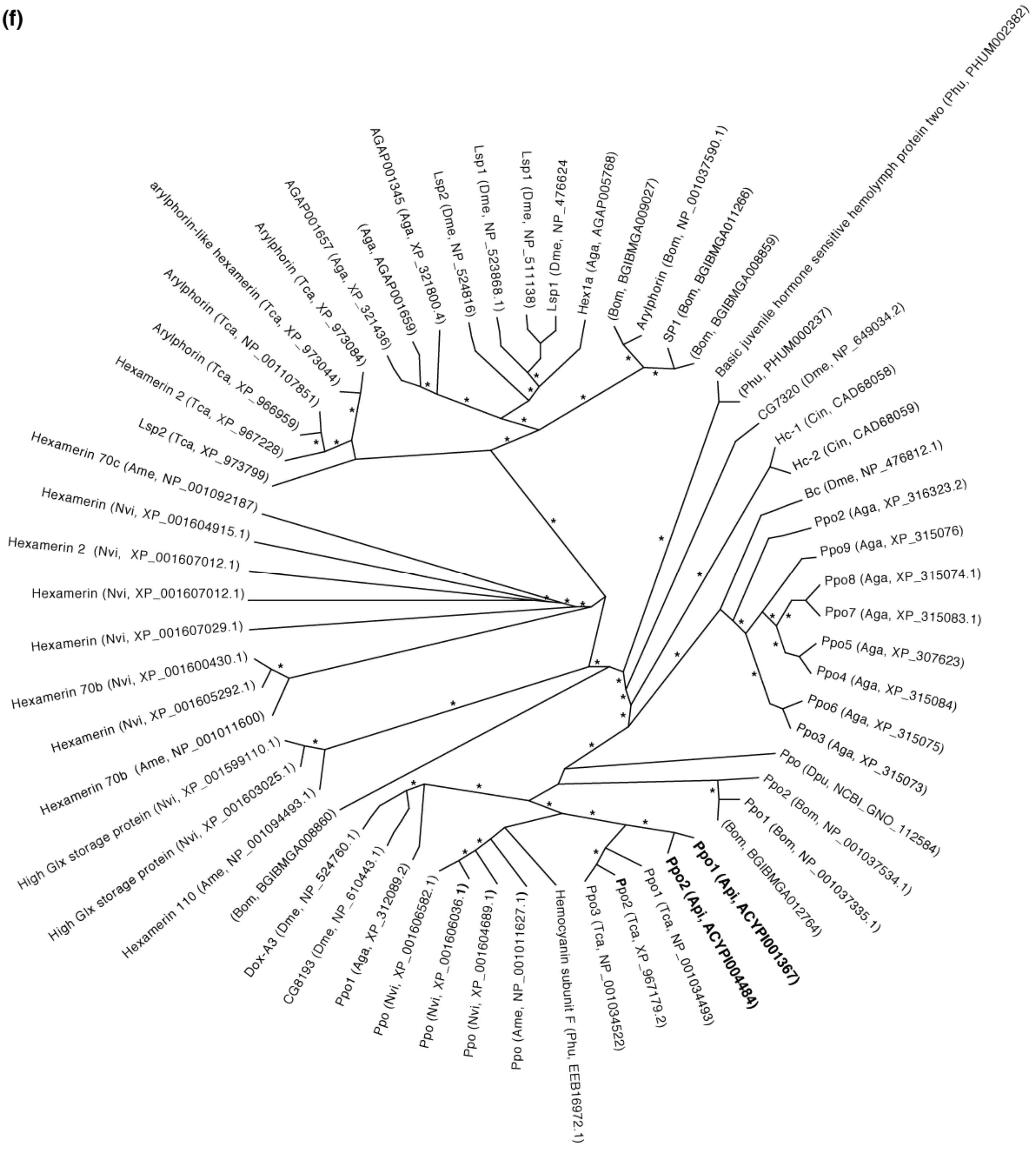

Basic juvenile hormone sensitive hemolymph protein two (Phu, PHUM002382)
(Phu, PHUM000237)
CG7320 (Dme, NP_649034.2)
Hc-1 (Cin, CAD68058)
Hc-2 (Cin, CAD68059)
Bc (Dme, NP_476812.1)
Ppo2 (Aga, XP_316323.2)
Ppo9 (Aga, XP_315076)
Ppo8 (Aga, XP_315074.1)
Ppo7 (Aga, XP_315083.1)
Ppo5 (Aga, XP_307623)
Ppo4 (Aga, XP_315084)
Ppo6 (Aga, XP_315075)
Ppo3 (Aga, XP_315073)
Ppo (Dpu, NCBI_GNO_112584)
Ppo2 (Bom, NP_001037534.1)
Ppo1 (Bom, NP_001037335.1)
(Bom, BGIBMGA012764)
Ppo1 (Api, ACYPI001367)
Ppo2 (Api, ACYPI004484)
Ppo1 (Tca, NP_001034493)
Ppo2 (Tca, XP_967179.2)
Ppo3 (Tca, NP_001034522)
Hemocyanin subunit F (Phu, EEB16972.1)
Ppo (Ame, NP_001011627.1)
Ppo (Nvi, XP_001604689.1)
Ppo (Nvi, XP_001606036.1)
Ppo (Nvi, XP_001606582.1)
Ppo1 (Aga, XP_312089.2)
CG8193 (Dme, NP_610443.1)
Dox-A3 (Dme, NP_524760.1)
(Bom, BGIBMGA008860)
Hexamerin 110 (Ame, NP_001094493.1)
High Glx storage protein (Nvi, XP_001603025.1)
High Glx storage protein (Nvi, XP_001599110.1)
Hexamerin 70b (Ame, NP_001011600)
Hexamerin (Nvi, XP_001605292.1)
Hexamerin 70b (Nvi, XP_001600430.1)
Hexamerin (Nvi, XP_001607029.1)
Hexamerin (Nvi, XP_001607012.1)
Hexamerin 2 (Nvi, XP_001607012.1)
Hexamerin (Nvi, XP_001604915.1)
Hexamerin 70c (Ame, NP_001092187)
Lsp2 (Tca, XP_973799)
Hexamerin 2 (Tca, XP_967228)
Arylphorin (Tca, XP_966959)
Arylphorin (Tca, NP_001107851)
arylphorin-like hexamerin (Tca, XP_973044)
Arylphorin (Tca, XP_973084)
AGAP001657 (Aga, XP_321436)
(Aga, AGAP001659)
AGAP001345 (Aga, XP_321800.4)
Lsp2 (Dme, NP_524816)
Lsp1 (Dme, NP_523868.1)
Lsp1 (Dme, NP_511138)
Lsp1 (Dme, NP_476624
Hex1a (Aga, AGAP005768)
(Bom, BGIBMGA009027)
Arylphorin (Bom, NP_001037590.1)
SP1 (Bom, BGIBMGA011266)
(Bom, BGIBMGA008859)

(g)

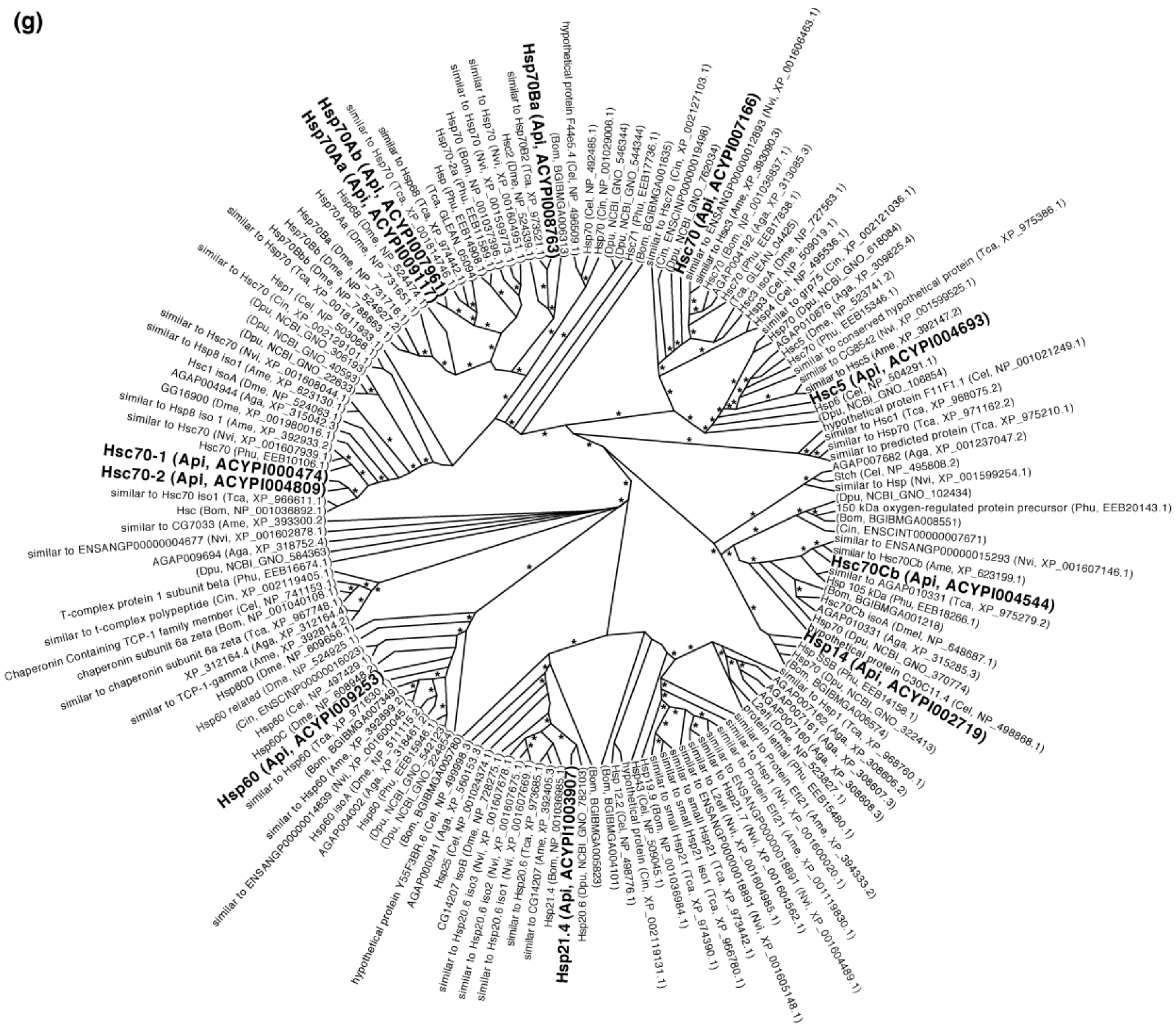

Hsp70Ab (Api, ACYPI007961)
Hsp70Aa (Api, ACYPI009117)
Hsp70Ba (Api, ACYPI008763)
Hsc70 (Api, ACYPI007166)
Hsc5 (Api, ACYPI004693)
Hsc70-1 (Api, ACYPI000474)
Hsc70-2 (Api, ACYPI004809)
similar to Hsc70 iso1 (Tca, XP_966611.1)
Hsc (Bom, NP_001036892.1)
similar to CG7033 (Ame, XP_393300.2)
similar to ENSANGP00000004677 (Nvi, XP_001602878.1)
AGAP009694 (Aga, XP_318752.4)
(Dpu, NCBI_GNO_584363)
T-complex protein 1 subunit beta (Phu, EEB16674.1)
similar to Hsp (Nvi, XP_001599254.1)
(Dpu, NCBI_GNO_102434)
150 kDa oxygen-regulated protein precursor (Phu, EEB20143.1)
(Bom, BGIBMGA008551)
(Cin, ENSCINT00000007671)
Stch (Cel, NP_495808.2)
AGAP007682 (Aga, XP_001237047.2)
Hsc70Cb (Api, ACYPI004544)
Hsp14 (Api, ACYPI002719)
Hsp60 (Api, ACYPI009253)
Hsp21.4 (Api, ACYPI003907)

Figure S2. **Alignments of putative antimicrobial peptides megourin and penaeidin. (**a) Putative pea aphid megourin (pea_aphid) aligned with 3 megourins of the aphid *Megoura viciae* (MEGVI). (b) Putative pea aphid penaeidin (ACYPI37769-PA) aligned with penaeidins from the shrimp *Penaeus vannamei* (PENVA) and *Penaeus (Litopenaeus) setiferus* (LITS).

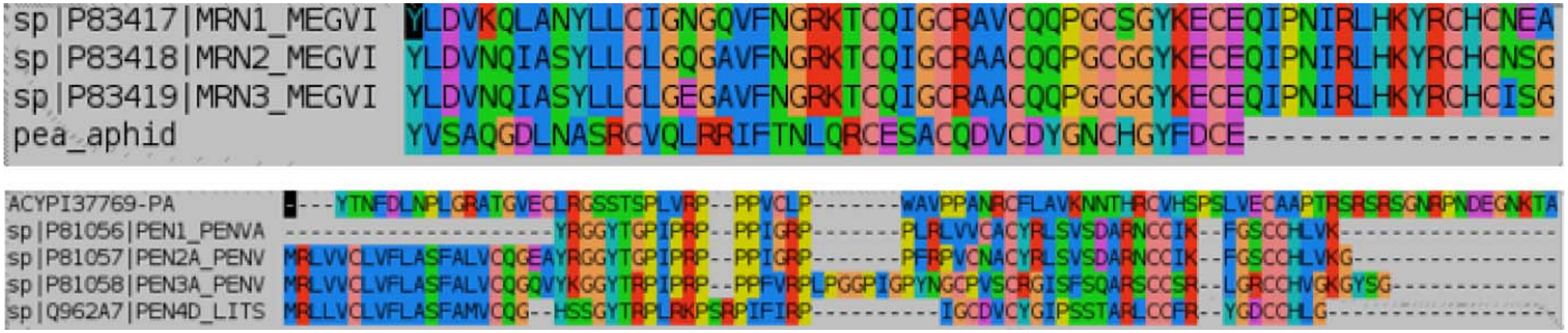

Figure S3. Survival curves for experimental infections associated with the qPCR study. (a) In the bacterial feeding experiment, 30 aphids per condition were fed for 20hrs on artificial diet containing bacteria or on control diet and then transferred to plants and monitored for survival. Though survival data for the bacterial stabbing experiment, in which aphids were stabbed with bacteria-contaminated needles, was not collected daily, after two days, overall survival was similar to the feeding trials, with 100% survival of control aphids, 80% survival of *E. coli*-stabbed aphids, 30% survival of Gram-positive pathogen-stabbed aphids, and 0% survival of Gram-negative pathogen-stabbed aphids (n = 10 per condition). (b) In the fungal shower experiment, 22 aphids were exposed to a shower of fungal spores for two hours and then transferred to plants. Control aphids were not exposed. (c) In the viral stabbing experiment, 20 aphids were stabbed with a virus-contaminated needle and then transferred to plants. Control aphids were not stabbed.

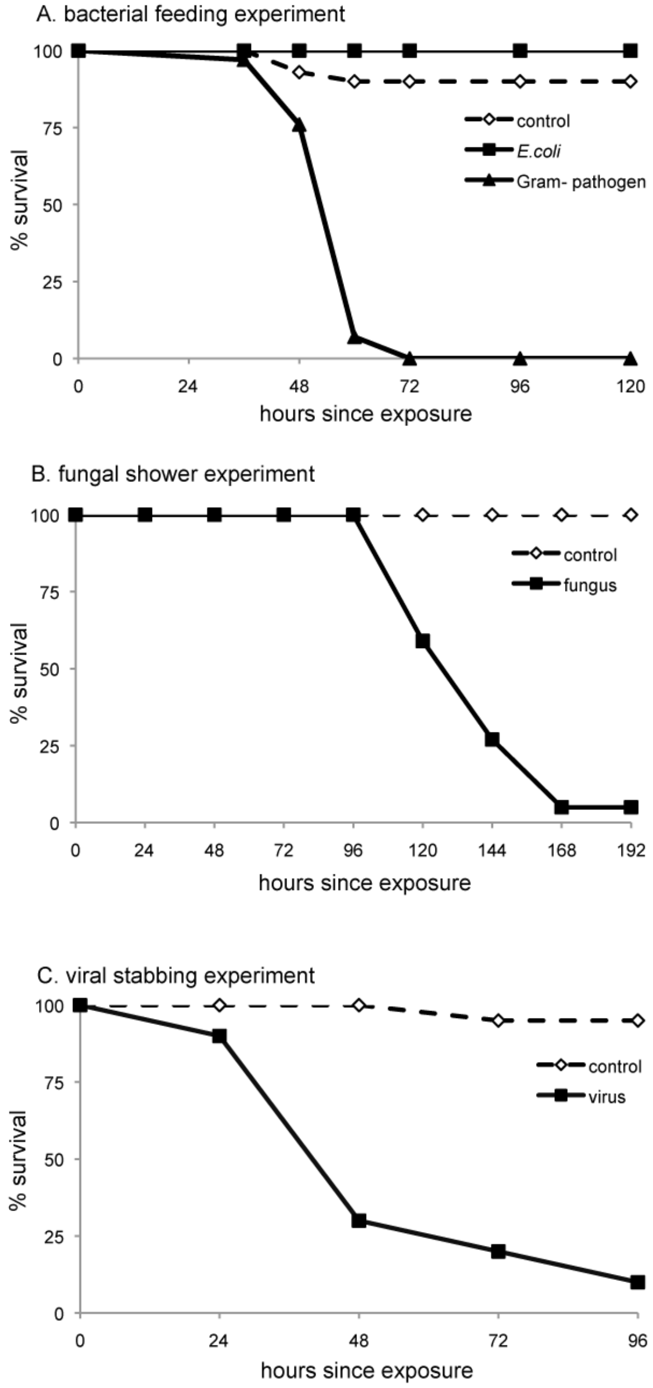

Supplement: Additional file 1 — Table S1: pea aphid immune and stress gene list. Table S2: samples for quantitative PCR expression study. Table S3: primers for quantitative PCR expression study. Table S4: relative expression of recognition and signaling genes. Table S5: relative expression of response genes. Table S6: gut EST library statistics. Table S7: list of selected ESTs from the subtracted library. Figure S1: maximum likelihood phylogenies of selected immune and stress gene families. Figure S2: alignments of putative antimicrobial peptides megourin and penaeidin. Figure S3: survival curves for experimental infections associated with quantitative PCR study. [file gb-2010-11-2-r21-S1.pdf]
